# Integrated chemometric evaluation of morphological traits, chemical composition, and hepatotoxicity of *Polygonum multiflorum*

**DOI:** 10.3389/fphar.2025.1666300

**Published:** 2025-10-23

**Authors:** Yanyi Li, Qi Wang, Yanrong Yang, Xiaobin Huang, Yujie Zhang

**Affiliations:** ^1^ School of Chinese Materia Medica, Beijing University of Chinese Medicine, Beijing, China; ^2^ Institute of Chinese Traditional Medicine and Ethnic Medicine, National Institutes for Food and Drug Control, Beijing, China; ^3^ School of Functional Food and Wine, Shenyang Pharmaceutical University, Shenyang, Liaoning, China

**Keywords:** *Polygonum multiflorum*, multi-index comprehensive evaluation model, chemometrics, component analysis, morphological traits, hepatotoxic constituents

## Abstract

**Ethnopharmacological relevance:**

The toxicity of *Polygonum multiflorum* (PM) is widely recognized. However, its toxic constituents and the mechanisms underlying their interactions remain insufficiently characterized.

**Aim:**

This study aims to establish a new evaluation model that integrates multiparameter chemometric analysis with combined statistical approaches to assess and monitor the toxicity of PM. The multi-index framework included crude drug morphological traits, main chemical constituents (anthraquinones, stilbene glycosides, flavonoids and phenols), and hepatotoxicity across different PM batches.

**Materials and methods:**

An ultra-performance liquid chromatography–electrospray ionization–triple-quadrupole tandem mass spectrometry (UPLC-ESI-QqQ-MS/MS) method was first developed to quantify 26 compounds in 61 batches of commercial PM samples. Chemometric analyses, including principal component analysis (PCA) and hierarchical cluster analysis (HCA), were then applied to classify the samples into four groups based on chemical composition. Hepatotoxicity comparisons among these groups were conducted using the Cell Counting Kit-8 assay in HepaRG cells. Subsequently, toxicity-related morphological traits were investigated by quantifying chromaticity values of crude drugs from groups with distinct toxicity profiles. Two key between-group toxicity variables were identified through chemometric analyses, including orthogonal partial least squares discriminant analysis (OPLS-DA) and *t*-tests. Systematic correlations between toxicity-related morphological traits and chemical composition were then established. Finally, emodin-8-*O*-β-*D*-glucoside (EG) and 2,3,5,4′-tetrahydroxystilbene-2-*O*-β-*D*-glucopyranoside (THSG), the two most abundant and distinctive components, were co-administered to explore potential toxic interactions.

**Results:**

The established chemical profiling library of PM demonstrated a strong correlation with hepatocytotoxicity. White decoction pieces of PM were found to be safer, while THSG and EG were identified as potential hepatotoxic constituents. Moreover, THSG enhanced the hepatotoxicity of EG, indicating a synergistic effect.

**Conclusion:**

The proposed model offers a novel framework linking morphological traits, chemical composition, and hepatotoxicity of PM, offering a reference for the safety evaluation of other medicinal herbs.

## 1 Introduction

*Polygonum multiflorum* Thunb. is a climbing plant whose root, commonly known as *Polygonum multiflorum* (PM), has long been valued for its nutritional and medicinal properties. It originated in East Asia and parts of North America, and it was first documented during the Tang dynasty in China for its high edible and therapeutic value (Committee, 2020; Lin et al., 2015; Teka et al., 2021). In traditional Chinese medicine (TCM), PM is a famous tonic, widely used for detoxification, intestinal moistening, and stool regulation (Committee, 2020). Modern pharmacological studies have demonstrated that PM contains anthraquinone derivatives, among which rhein can effectively promote intestinal peristalsis and exerts a significant laxative effect. In addition, the main active constituent of PM, 2,3,5,4′-tetrahydroxystilbene-2-*O*-β-*D*-glucopyranoside (THSG), has been shown to reduce serum total cholesterol, low-density lipoprotein cholesterol, and the atherosclerotic index, thereby lowering blood lipid levels and exerting anti-atherosclerotic effects (Li F. et al., 2020). In addition, clinical applications and contemporary pharmacological studies have confirmed that PM exhibits antioxidative, antitumor, antifatigue, immunoenhancing, and antibacterial properties, which contributes to its health benefits, particularly for middle-aged and elderly individuals (Sun et al., 2021; Xue et al., 2020; Wang Y. et al., 2023).

However, it is important to note that while PM has a long history of use in traditional medicine, its clinical efficacy is sufficiently supported by modern evidence-based studies. Moreover, a systematic benefit-risk assessment for PM is currently lacking, due to the variability in chemical composition and inter-component interactions (Huang et al., 2024; Wang et al., 2022; Liu et al., 2018; Li R. L. et al., 2020). This underscores the urgency of conducting rigorous clinical trials to validate its therapeutic claims and to establish a safety profile relative to its benefits. The earliest documented case of PM-induced hepatotoxicity dates back to 1988 (Jia et al., 2022), and since then, reports of liver injury associated with PM have increased in China and other countries (Hinshaw, 1996; Park et al., 2001; Cárdenas et al., 2006; Teschke et al., 2014; Lei et al., 2015). Therefore, extensive research has been conducted to identify the hepatotoxic constituents of PM and elucidate their underlying mechanisms. PM contains several classes of chemical compounds, including anthraquinones, stilbene glycosides, flavonoids and phenols. Among them, stilbene glycosides, such as THSG and *cis*-2,3,5,4′-tetrahydroxystilbene-2-*O*-β-*D*-glucopyranoside (*cis*-THSG), are the most abundant, accounting for approximately 1.00% of the total concentration of compounds in PM. In contrast, anthraquinones (such as emodin-8-*O*-β-*D*-glucoside [EG] and physcion), flavonoids (such as catechins and epicatechins), and phenolic compounds (such as gallic acid and *p*-hydroxybenzoic acid) are present at lower levels, representing 0.15%, 0.08%, and 0.02%, respectively (Li et al., 2022b, Li et al., 2023). Multiple pharmacological studies have indicated that anthraquinones possess hepatotoxic potential. For example, Wang et al. (2020) reported that in rats, emodin, rheinic acid, EG, physcion, and emodin-type monoanthone exhibit significant hepatotoxicity in liver microtissues. Similarly, Park et al. (2023) demonstrated that emodin increases caspase-3 expression and induces dose-dependent cytotoxicity in HepG2 cells. In contrast, studies on the hepatotoxicity of stilbene glycosides have mainly focused on *cis*-THSG, while relatively few investigations have addressed the hepatotoxicity of THSG itself (Yang et al., 2020). Liu et al. (2024) and Meng et al. (2021) suggested that *cis*-THSG induces immune-mediated liver injury by inhibiting peroxisome proliferator-activated receptor-γ (PPAR-γ). Moreover, a clinical study analyzing the blood of patients with adverse reactions to PM preparations detected only EM and THSG (Panis et al., 2005). Although THSG has recently been found to promote the absorption of anthraquinone compounds while inhibiting their metabolism, thereby exacerbating liver injury (Yu et al., 2017, Li D. et al., 2022), detailed information on dose–response relationships and the synergistic or antagonistic interactions among PM constituents remains limited.

There are significant differences in the compositional ratios and morphological traits of commercially available PM, which are primarily due to variations in growth environments and harvest cycles. However, the relationships among morphological traits, chemical composition, and hepatotoxicity have not been systematically investigated. A previous study by our research group demonstrated that the chemical composition of PM is closely related to its morphological characteristics, with the concentrations of main chemical constituents in PM decoction pieces varying according to different traits (Wang et al., 2024). Given the complex composition of PM, interactions among anthraquinones, stilbene glycosides, flavonoids and phenols, and other constituents may influence the occurrence of liver injury. Therefore, this study investigates the correlations among the morphological traits, chemical composition, and hepatotoxicity of commercial PM, and further identifies the colors and constituents most strongly associated with hepatotoxic potential. To achieve this, targeted chemical analysis, statistical processing, and toxicological assays were employed. In addition, the identified toxic constituents were analyzed for synergistic, additive, or antagonistic effects on hepatotoxicity.

To address variability in composition and the unclear links among morphological traits, chemical composition, and hepatotoxicity, the concentrations of 26 compounds in 61 batches of commercial PM samples with different morphological traits were determined using ultra-performance liquid chromatography–electrospray ionization–triple-quadrupole tandem mass spectrometry (UPLC-ESI-QqQ-MS/MS). Hierarchical cluster analysis (HCA) was then applied to group PM samples with similar compositions, and principal component analysis (PCA) was used to validate the accuracy of the grouping, ultimately classifying all 61 batches of PM samples into four distinct groups. To investigate the relationship between the chemical composition of PM and hepatotoxicity, cytotoxicity assays were conducted using the HepaRG cell line. The results showed that samples within the same group exhibited comparable cytotoxicity, while those from different groups displayed clearly distinguishable levels of cytotoxicity, which were categorized into high, medium, or low levels. Subsequently, stereo fluorescence microscopy was used to examine toxicity-related chromaticity features, while orthogonal partial least squares discriminant analysis (OPLS-DA) and one-sample *t*-tests were applied to identify differential constituents affecting intergroup toxicity differences. Finally, based on the hepatotoxic compounds identified, a “One-belt, One-line” drug combination model was employed to investigate how two toxic constituents inhibited liver cell proliferation. Overall, this study elucidates the correlations among the morphological traits, chemical composition, and hepatotoxicity of PM. These findings not only provide a scientific basis for improving patient and consumer safety but also support the quality control and regulatory oversight of herbal medicinal products.

## 2 Materials and methods

### 2.1 Chemicals and reagents

The standards of the compounds used for quantification and method validation are shown in Supplementary Table S1. Methanol, acetonitrile, and formic acid (LC-MS grade) were obtained from Honeywell (United States). Ultrapure water with a resistivity of 18.2 MΩ cm was produced using the Milli-Q IQ 7000 water cleansing system (Merck Millipore, United States). All additional analytical-grade reagents and chemicals were purchased from Sinopharm Group Chemical Reagents (China). Stock solutions of the standard compounds were prepared in methanol at a concentration of 1 g/L and then stored at −20 °C. Calibration curves were subsequently constructed using these solutions.

William’s Medium E (WME), acetaminophen (APAP), and dimethyl sulfoxide (DMSO) were obtained from Sigma-Aldrich (United States). Fetal bovine serum (FBS), phosphate-buffered saline (PBS), 0.05% trypsin–EDTA solution, penicillin (10,000 μg/mL), and streptomycin (10,000 μg/mL) were purchased from HyClone (United States). The Cell Counting Kit-8 (CCK-8) was obtained from Dojindo Molecular Technologies (China). THSG and E.G., were dissolved in DMSO at concentrations of 700 mg/mL and 200 mg/mL, respectively, and then diluted in the medium. The final concentration of DMSO in the medium was maintained below 0.1% (v/v) (Yu et al., 2017; Lee et al., 2017).

### 2.2 Sample preparation

**FIGURE 1:**
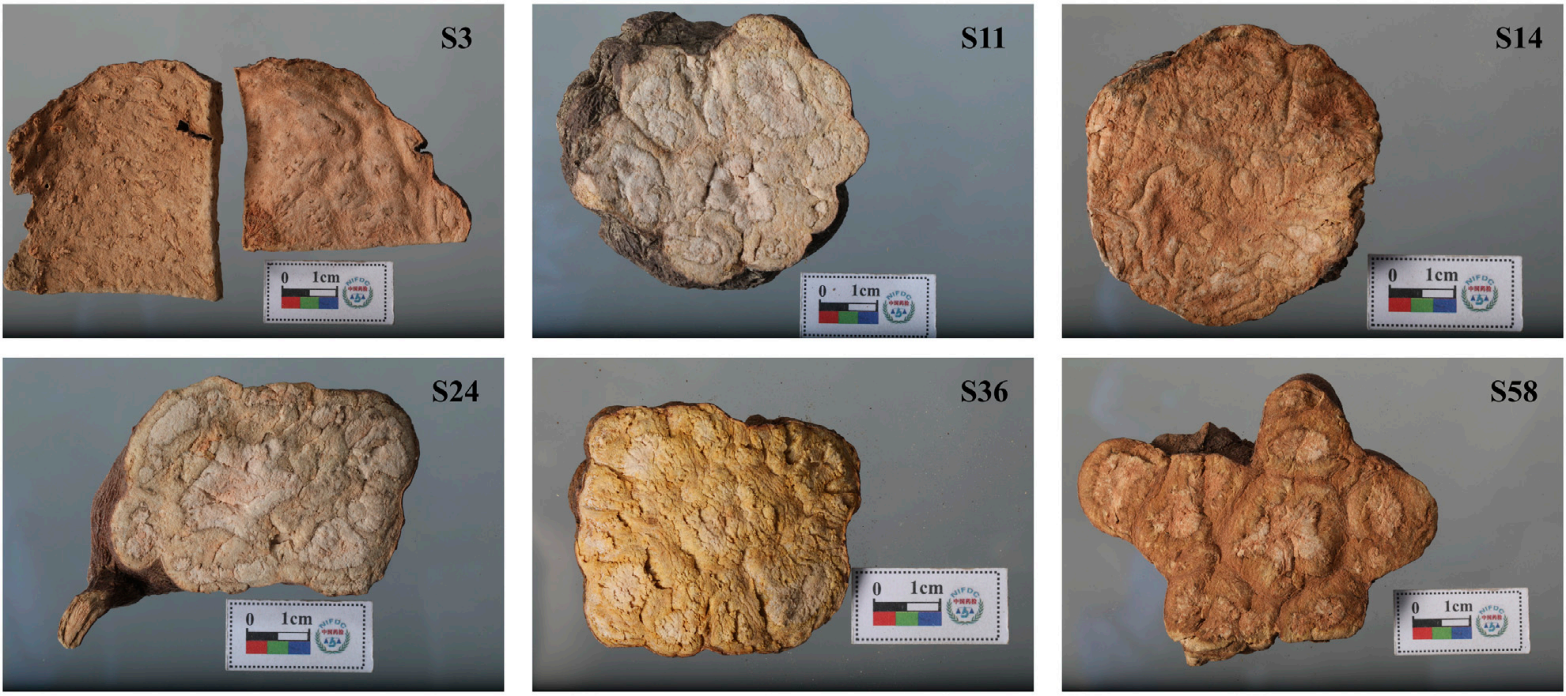
Morphological traits of collected commercial PM samples.

According to the 2020 edition of the *Pharmacopoeia of the People’s Republic of China* (ChP), qualified PM decoction pieces are characterized by a red-brown or dark brown outer surface, a yellowish-brown or pale reddish-brown fractured surface, and a slightly astringent taste (Committee, 2020). They also exhibit 4–11 roundish abnormal vascular bundles in the bark, arranged in a ring and forming cloud-like patterns. In total, 61 batches of commercial PM samples were randomly collected from five regions in China: Yunnan (YN, *n* = 11), Anhui (AH, *n* = 10), Guangdong (GD, *n* = 10), Guizhou (GZ, *n* = 20), and Sichuan (SC, *n* = 10), as detailed in Supplementary Table S2. These samples displayed morphological variations in color, size, and decoction-piece specifications (Figure 1). All samples consisted of dried roots of *Polygonum multiflorum* Thunb., and their identity was confirmed by the China National Institutes for Food and Drug Control.

The UPLC-ESI-QqQ-MS/MS test solution was prepared as follows: the 61 batches of samples were ground into powder, sieved using a Chinese National Standard Sieve No. 4 (250 µm mesh), and weighed (1 g per sample). Each sample was transferred into a 100 mL stoppered conical flask, to which 50 mL of 70% ethanol (v/v) was added. The flask was weighed, and ultrasonic extraction was performed in a 30 °C water bath for 30 min. After the extract cooled to room temperature, the lost weight was replaced with 70% ethanol. The extract was filtered, and 25 mL of the filtrate was evaporated in a water bath. The residue was dissolved in 5 mL of 70% ethanol and filtered through a 0.22 µm microporous membrane to obtain the UPLC-ESI-QqQ-MS/MS stock solution. A 200 µL aliquot of the stock solution was diluted to 10 mL (i.e., 50-fold dilution) to prepare the working solution. The ultrasonic extraction method using 70% ethanol at 30 °C was selected based on our previous systematic optimization and validation (Lv et al., 2015; Yang et al., 2018; Zhang et al., 2020). This method consistently achieved efficiencies greater than 85% across all 26 target compounds, which included anthraquinones, stilbene glycosides, flavonoids and phenols. Furthermore, the analytes demonstrated stability under the applied conditions, with no significant degradation observed throughout the analysis (Li et al., 2022a).

The preparation of PM solutions for HepaRG cell assays followed the same procedure, involving ultrasonication in 50 mL of 70% ethanol and subsequent evaporation of 25 mL of the filtrate. The residue was dissolved in 0.5 mL of DMSO to obtain the test solution for cell-based studies. For administration in 96-well plates, the DMSO concentration was adjusted to ≤0.1%.

### 2.3 Quantitative analysis of major PM constituents

Using a previously validated UPLC-ESI-QqQ-MS/MS method, we quantified 26 compounds in PM, including anthraquinones, stilbene glycosides, flavonoids and phenols (Li et al., 2022a). This method involved comprehensive assessments of extraction recovery for all analytes as well as their stability throughout the analytical process, ensuring the reliability of the quantitative results. Concentrated and diluted PM solutions were analyzed using an LC-20AD system (Shimadzu, Japan), with an injection volume of 1 µL and a column temperature of 27 °C. The column was an ACQUITY UPLC™ HSS C_18_ column (100 mm × 2.1 mm, 1.8 μm), mobile phase A was 100% acetonitrile, and mobile phase B was a 0.2% formic acid solution. Gradient elution was performed as follows: 0–3.2 min, 10%–15% A; 3.2–6.5 min, 15% A; 6.5–9.7 min, 15%–26% A; 9.7–12 min, 26%–32% A; 12–15 min, 32%–36% A; 15–16.5 min, 36% A; 16.5–31 min, 36%–80% A; 31–35.9 min, 80% A; 35.9–36 min, 80%–10% A; and 36–41 min, 10% A. The flow rate was set at 0.4 mL/min.

Detection was conducted on an LCMS-8050-QqQ-mass-spectrometer (Shimadzu, Japan) operated in both positive and negative ionization modes, with the mode providing the highest compound clarity selected for quantification. The electrospray ionization (ESI) source parameters were set at 300 °C for temperature and 3,000 V for ion spray voltage. For each analyte, the two most abundant ions were selected as precursor ions, and the product ions with the strongest responses were identified through product-ion scanning. For quantification, the two strongest transitions (a quantifier and a qualifier) were selected, and their collision energies were optimized. The method was validated for trueness, repeatability, specificity, linearity, and sensitivity. Further details of the methodological validation are provided in our previous study (Li et al., 2022a).

### 2.4 Statistical analysis of quantitative results

Quantitative analysis of the chemical composition of each PM batch was performed using the LabSolutions browser (Shimadzu, version 5). Compound concentrations were determined by comparing analyte peak areas with those of corresponding standards, and the concentrations of the 26 compounds were expressed as mean values from three replicates. For chemometric analysis, the preprocessed data were exported to OriginPro 2021 for normalization, and the normalized model was validated for interpretability using variable importance in projection (VIP) and *Corr.Coeffs* values. Then, heatmaps combined with hierarchical cluster analysis (HCA) were generated using Tbtools to visualize sample distribution patterns. PCA with seven-fold cross-validation was performed using SIMCA-P 14.1 to verify classification reliability and to identify discriminant metabolites associated with grouping (Yin et al., 2024; Ji et al., 2024). To assess the influence of geographic origin on PM chemical composition, one-way analysis of variance (ANOVA) was conducted using OriginPro 2021. The total concentration of the 26 compounds in each sample was used as the dependent variable, and the five geographic origins (Yunnan, Anhui, Guangdong, Guizhou, and Sichuan) were set as the categorical independent variable.

### 2.5 Cytotoxicity assays of PM in HepaRG cells

The HepaRG cell line was obtained from Thermo Fisher Scientific (United States), and cells from the 12th to 13th generations were used in the experiments. Cells were cultured in WME supplemented with 10% fetal bovine serum (FBS, v/v) and 1% penicillin–streptomycin (v/v), and maintained at 37 °C in a humidified atmosphere containing 5% CO_2_. Cells in the logarithmic growth phase were used for subsequent experiments. To evaluate the potential hepatotoxicity of compliant PM samples, 29 batches were systematically selected from three groups of qualified samples (*n* = 9 in Group B, *n* = 11 in Group C, and *n* = 9 in Group D). In addition, two samples from Group A (S1 and S3), which did not meet the pharmacopeial standard for THSG content (<1%), were included in the cytotoxicity assays to provide a more comprehensive understanding of the relationship between compositional deviation and toxicity. The selection of samples across groups considered both geographical origin and morphological traits, covering five provinces (Yunnan, Anhui, Guangdong, Guizhou, and Sichuan) and three chromaticity categories (white, yellow, and brown), thereby ensuring representativeness, typicality, and randomness.

Cell viability was assessed using the CCK-8 assay (Wang T. K. et al., 2023). Cells in the logarithmic growth phase were seeded into 96-well plates at a density of 8 × 10^3^ cells/well and incubated for 24 h. To minimize edge effects, the outer wells of each plate were filled with PBS. Cells were then exposed to serum-free medium containing different concentrations of PM extracts (1,000, 100, 10, and 1 μg/mL) for 72 h. Following exposure, 100 µL of CCK8 solution in serum-free medium was added to each well, and cells were incubated at 37 °C for 2 h. Absorbance was measured at 450 nm using a Biotek Epoch microplate reader (Agilent, United States). Each treatment group included four replicates. Cell viability was calculated as (dosing − blank)/(control − blank). APAP was used as a positive control for hepatotoxicity. It was dissolved in DMSO and diluted in WME to final concentrations of 1–20 mM, with the DMSO concentration maintained below 0.1% (v/v) (Yokoyama et al., 2018; Van den Eede et al., 2015). Cells were treated with APAP for 72 h under the same conditions as PM samples. The half-maximal inhibitory concentration (IC_50_) values of the 29 p.m. batches were determined, and significant differences were analyzed using one-way ANOVA with Duncan’s *post hoc* test. Statistical significance was set at *p* < 0.05.

### 2.6 Statistical analysis of chromaticity values related to toxicity

Previous studies have shown that the color characteristics of herbal medicines provide insight into their internal composition (Zhang et al., 2023; Liu et al., 2018). On this basis, 15 batches of PM samples (*n* = 5 per group) that passed the content test were selected from the three groups to study the correlation between the chromaticity and toxicity of decoction pieces. A stereoscopic fluorescence microscope was used to capture images of four anatomical regions (the cork, phloem, central xylem, and heteromorphic vascular bundles) from both the front and back surfaces of PM decoction pieces. Compared to colorimeters and spectrophotometers, this microscope provides enhanced magnification of morphological characteristics. To ensure reproducibility and account for sample heterogeneity, five different sites were measured for each anatomical region. The images were imported into Photoshop CS6, where chromaticity indices (R, G, B, L*, a*, and b*) were extracted. For each anatomical region, the mean chromaticity value was calculated from the five measured sites, resulting in four averaged chromaticity values for each surface. Subsequently, mean values from all 40 measurements per sample (4 regions × 5 sites × 2 surfaces) were used to calculate the overall color parameter according to the formula E*ab = [L*2+a*2 + b*2]^1/2^. HCA was then performed using OriginPro 2021 to explore the correlations between the chemical-composition-based toxicity classifications of PM and the chromaticity values of different anatomical regions of the decoction pieces. To verify the above correlation, the RGB values were normalized using the following equations: Normalized R = r/(r + g + b), Normalized G = g/(r + g + b), Normalized B = b/(r + g + b). The normalized values were subsequently subjected to regression analysis against the IC_50_ concentrations obtained from the hepatocellular cytotoxicity assays. This approach follows the normalization strategy described by Guo et al. (2020), who applied RGB values to evaluate the vegetation index of maize leaves and demonstrated a strong linear relationship between them.

### 2.7 Identification of hepatotoxic compounds

The preceding analyses demonstrated that hepatocytotoxicity data were correlated with the quantitative profiles of PM components. To further investigate this relationship, an OPLS-DA model was constructed using SIMCA-P 14.1 software to maximize intergroup separation and identify the key compounds responsible for these differences (Hou et al., 2024; Zhou et al., 2024). The validity of the chemometric model was evaluated using the R^*2*^ and Q^*2*^ parameters, with values greater than 0.5 indicating good model quality, and values approaching 1 reflecting excellent model performance. Compounds were ranked by their VIP values, and those with VIP ≥1 were considered to contribute significantly to group classification. To minimize overfitting, the OPLS-DA models were rigorously validated through a 200× permutation test (Uttl et al., 2019). In this test, the order of the Y-variable (sample class) was randomly permuted, and a new model was generated for each permutation. A model was considered valid if the R^2^ and Q^2^ values of the permuted models were significantly lower than those of the original model, and if the regression line of Q^2^ against the correlation coefficient between the original and permuted Y-variables displayed a negative intercept. For further chemometric verification, the quantitative data from different PM groups were exported into Excel for one-sample *t*-tests. To mitigate the risk of false positives in marker selection, *p*-values from the *t*-tests were adjusted using the False Discovery Rate (FDR) correction according to the Benjamini–Hochberg method. Compounds with VIP >1 and an FDR-adjusted *q*-values <0.05 were considered statistically significant differential constituents.

### 2.8 Determination of Chou–Talalay combination index (CI) values

Stock solutions of potentially toxic compounds were prepared in DMSO, and hepatocellular cytotoxicity assays were conducted at physiologically relevant concentrations (30–700 μg/mL for THSG and 1–80 μg/mL for E.G.,), as described in Section 2.5. These ranges were selected based on plasma and tissue concentrations reported in previous *in vivo* pharmacokinetic studies following PM extract administration, particularly those associated with hepatotoxic outcomes (Yu et al., 2017, Li D. et al., 2022). The “One-belt, One-line” method, a novel drug combination assessment approach analogous to the Chou–Talalay method, was applied to evaluate compound interactions. In this approach, the expected additive effect of a multi-drug combination is displayed as a curved band on a two-dimensional coordinate plot, while the actual dose–response relationship of the tested combination is represented as a curve within the same space. The resulting alignment is referred to as the “One-belt, One-line” phenomenon (Yuan and Chen, 2019). Using Prism 5.02 and OriginPro 2021, dose–response curves for individual compounds and their combinations were generated, and CI values were calculated to evaluate interactions between potential toxic compounds. A CI value of 1 indicates an additive effect, a CI value <1 suggests synergy, and a CI value >1 indicates antagonism. Although initially developed for efficacy studies, the “One-belt, One-line” model can be extended to toxicological studies, as it is based on the same isobolographic principles as the Chou–Talalay method, which has been widely used in both efficacy and toxicity studies (Sriramulu et al., 2024; Wu et al., 2024).

## 3 Results

### 3.1 Quantification of 26 compounds in PM samples

**FIGURE 2:**
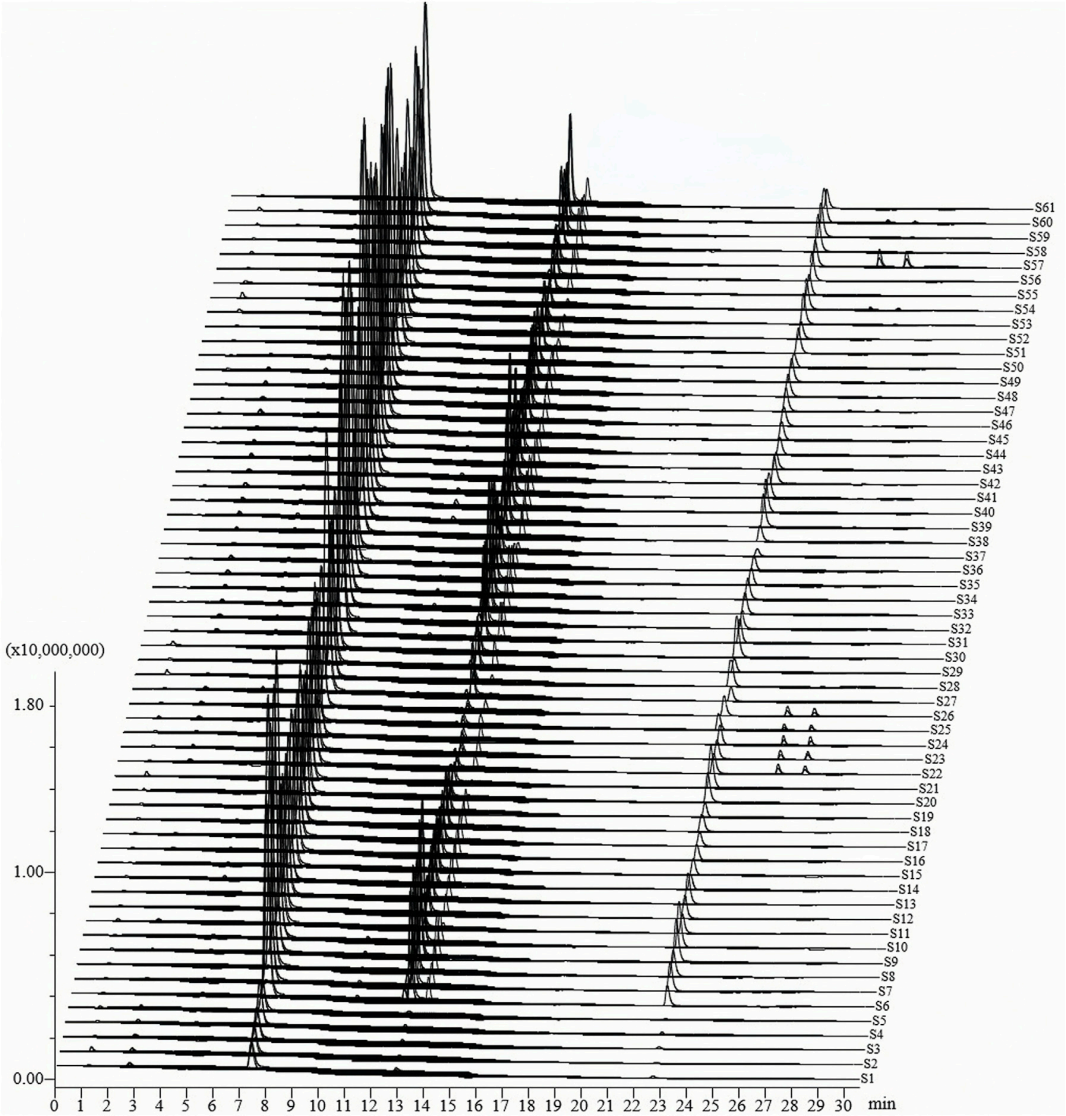
Quantification of 26 compounds in commercial PM samples using UPLC-ESI-QqQ-MS/MS method.

**FIGURE 3:**
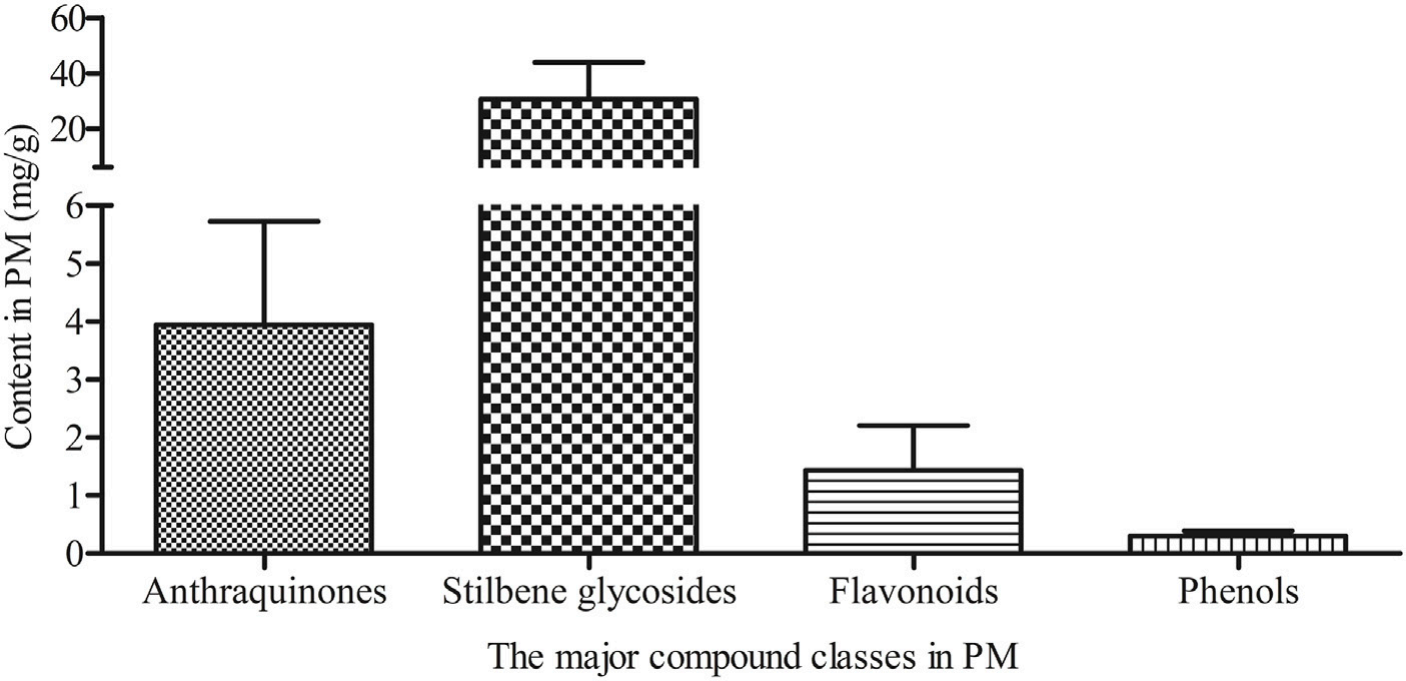
Average total contents of four major compound classes in commercial PM samples (*n* = 61).

Based on the literature review by Li et al. (2022a), 26 compounds were selected for quantitative analysis of PM samples. These compounds were required to be present in PM and have been reported in literature as being associated with toxic effects. Under identical quality control conditions, 12 anthraquinones, 5 stilbene glycosides, 4 flavonoids, and 5 phenols in test solutions from 61 p.m. batches were analyzed (Figure 2; Supplementary Tables S3–S5). The total concentrations of anthraquinones in PM samples ranged from 0.22 to 7.92 mg/g. Among them, emodin, physcion, physcion-8-glucoside, *trans*-emodin dianthrones, EG, and emodin-8-*O*-(6′-methylmalonyl)-glucopyranoside were the main anthraquinones, accounting for 94.27% of the total anthraquinone content (Supplementary Table S3). In contrast, rhein, emodin-6-glucoside, 1-methyl emodin, emodin-1-glucoside, *cis*-emodin dianthrones, and physcion-8-*O*-(6′-methylmalonyl)-glucopyranoside were the minor anthraquinones (Supplementary Table S3). Stilbene glycosides represented the most abundant compound class, with concentrations ranging from 4.35 to 59.43 mg/g. THSG was the dominant constituent, accounting for 96.24% of the total stilbene glycoside content (Supplementary Table S4). Meanwhile, 2,3,5,4′-tetrahydroxystilbene-2-*O*-(2″-*O*-feruloyl)-*β-D*-glucopyranoside, *cis*-2,3,5,4′-tetrahydroxystilbene-2-*O-*β-*D*-glucoyranoside, resveratrol, and polydatin were minor constituents, with average contributions of 1.74%, 0.50%, 0.26%, and 1.25%, respectively (Supplementary Table S4). Among the flavonoid and phenolic compounds, catechin, epicatechin, and torachrysone-8-*O*-glucoside exhibited average concentrations above 0.1 mg/g (Supplementary Table S5). A graphical summary of the average total contents of the four major compound classes (anthraquinones, stilbene glycosides, flavonoids and phenols) across all 61 batches is presented in Figure 3. This visualization clearly demonstrates that stilbene glycosides are the most abundant compound class in PM, followed by anthraquinones, consistent with the quantitative results described above.

### 3.2 Classification of PM samples based on compound profiles

According to the 2020 edition of the Chinese Pharmacopeia, the THSG content in PM should not be less than 1.0% (Committee, 2020). Based on this criterion, Group A samples (S1–S5) were classified as unqualified and excluded from subsequent hepatocellular cytotoxicity assays.

**FIGURE 4:**
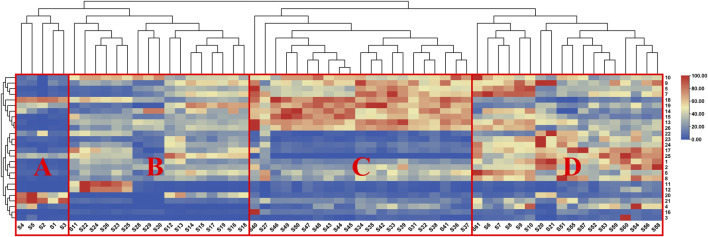
Heatmap clustering commercial PM samples into four groups **(A–D)** based on quantified compounds.

**FIGURE 5:**
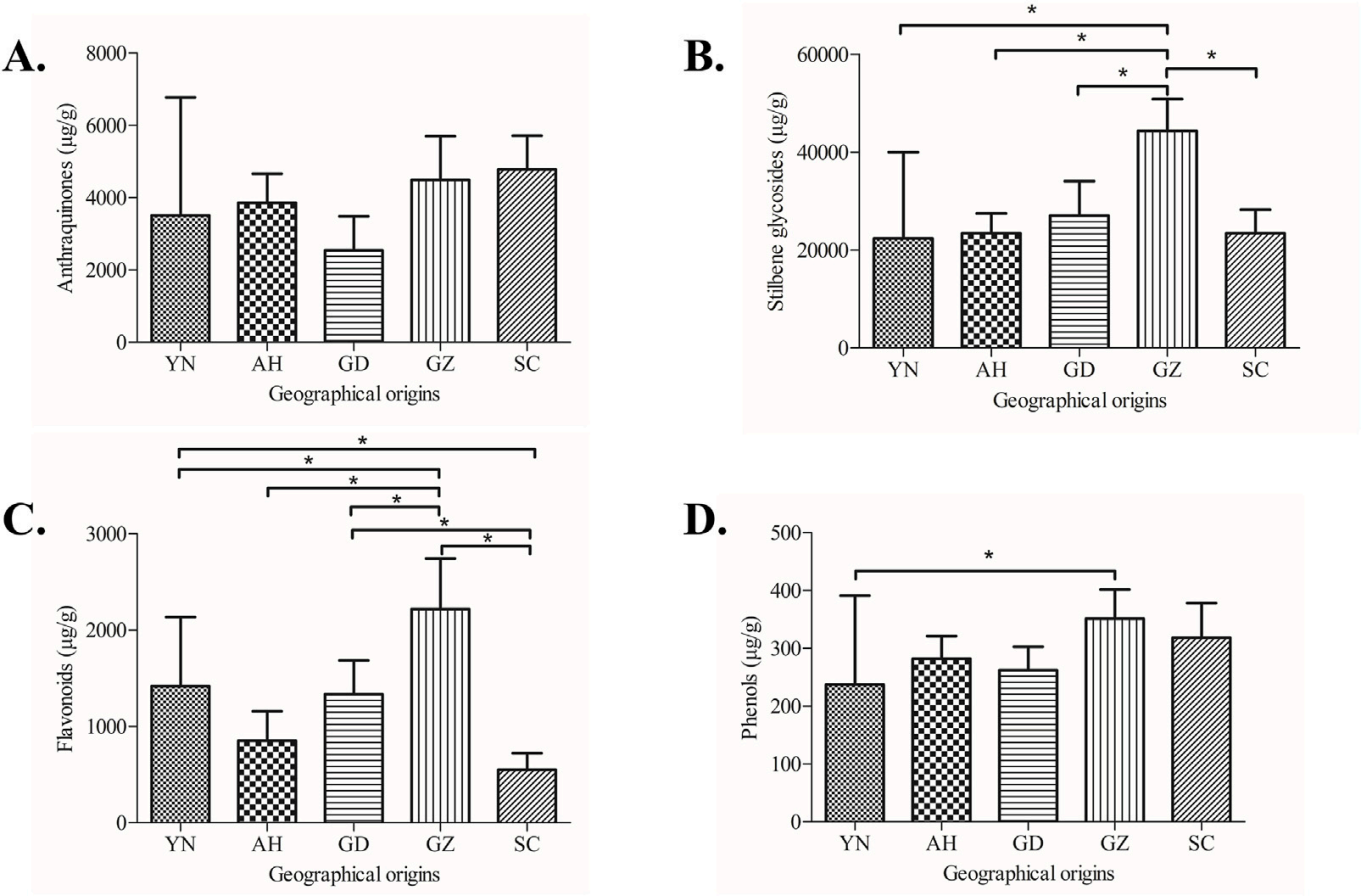
One-way ANOVA analysis of four major compound classes of commercial PM samples from five geographical origins: **(A)** Anthraquinones. **(B)** Stilbene glycosides. **(C)** Flavonoids. **(D)** Phenols. (YN = Yunnan [*n* = 11], AH = Anhui [*n* = 10], GD = Guangdong [*n* = 10], GZ = Guizhou [*n* = 20], SC = Sichuan [*n* = 10], *p < 0.01).

To explore similarities among PM samples from different batches, an HCA model was constructed using all 61 samples. The samples clustered into four distinct categories (Figure 4): Group A (S1–S5. unqualified THSG content), Group B (S11–S19, S22–S26, and S28–S30), Group C (S27 and S31–S50), and Group D (S6–S10, S20–S21, and S51–S61). There was no strong correlation between the chemical-composition-based classifications of PM samples and their geographic origins, suggesting that variations in morphological traits of PM are greater than those associated with geographical origin. One-way ANOVA further confirmed that the total content of the four major compound classes (anthraquinones, stilbene glycosides, flavonoids and phenols) in PM samples showed no significant difference compared to geographical origins. For anthraquinones (Figure 5A), pairwise comparisons between geographical areas showed no significant differences among the 10 pairs: AH vs. YN (*p* = 0.9893), GD vs. YN (*p* = 0.7472), GD vs. AH (*p* = 0.4768), GZ vs. YN (*p* = 0.5021), GZ vs. AH (*p* = 0.8462), SC vs. YN (*p* = 0.5419), GZ vs. GD (*p* = 0.0393), SC vs. AH (*p* = 0.8357), SC vs. GD (*p* = 0.069), SC vs. GZ (*p* = 0.9997). For stilbene glycosides (Figure 5B), there was a significant difference between GZ and YN (*p* < 0.01), GZ and AH (*p* < 0.01), GZ and GD (*p* < 0.01), SC and GZ (*p* < 0.01), while the other 6 pairs showed no significant differences: AH vs. YN (*p* = 0.9989), GD vs. YN (*p* = 0.8838), GD vs. AH (*p* = 0.9649), SC vs. YN (*p* = 0.9937), SC vs. AH (*p* = 0.9999), SC vs. GD (*p* = 0.9865). For flavonoids (Figure 5C), there was no significant difference between three pairs of regions, including AH vs. YN (*p* = 0.0163), GD vs. YN (*p* = 0.8619), GD vs. AH (*p* = 0.1911), SC vs. AH (*p* = 0.2933). Similarly, for phenols (Figure 5D), except for significant difference between GZ vs. YN (*p* = 0.0041), there were no significant differences in the other 9 pairs: AH vs. YN (*p* = 0.7092), GD vs. YN (*p* = 0.8511), GD vs. AH (*p* = 0.9989), GZ vs. AH (*p* = 0.2064), GZ vs. GD (*p* = 0.1127), SC vs. YN (*p* = 0.2085), SC vs. AH (*p* = 0.9061), SC vs. GD (*p* = 0.789), SC vs. GZ (*p* = 0.7721). Overall, there was no significant difference between the geographical origins and compound content of the vast majority of samples, further indicating that the chemical grouping of PM was not related to its geographical origin.

Heatmaps were then generated to visualize compositional differences among the four sample groups. This approach highlighted several major quantitative composition patterns and facilitated comparisons of differential compounds among groups (Wang Q. et al., 2023; Yin et al., 2024; Ziółkowska et al., 2016).

**FIGURE 6:**
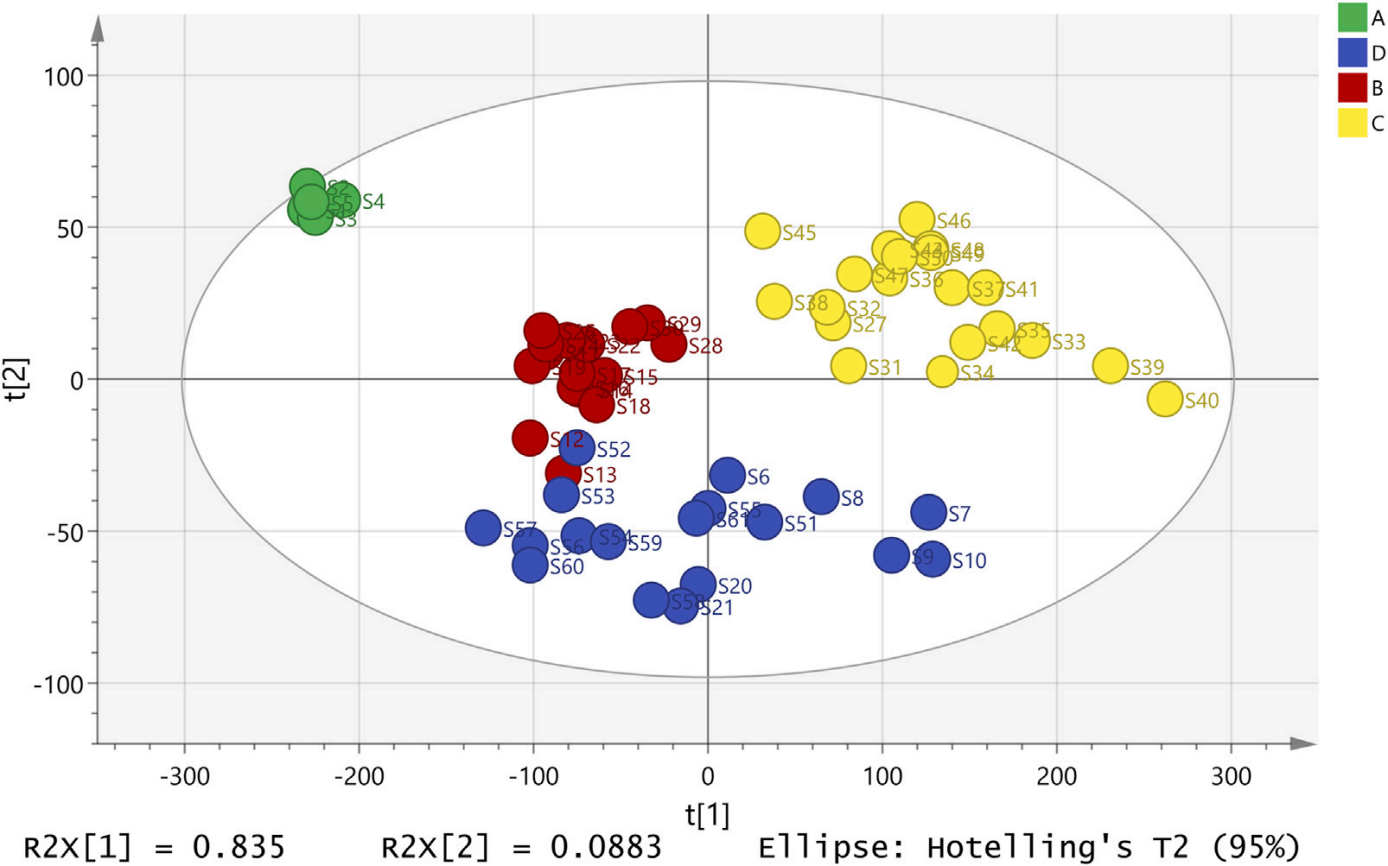
A PCA score plot showing the separation of different groups of commercial PM samples.

To validate the reliability of the classification, an unsupervised pattern recognition approach was applied (Luo et al., 2024). The PCA score plot (Figure 6) revealed that the first (PC1) and second (PC2) principal components accounted for 83.50% and 8.83% of the total variance, with a cumulative explanatory power R^2^X (cum) of 92.40%. Between Groups B and D was observed, suggesting shared chemical characteristics, such as comparable levels of flavonoids and phenols (Supplementary Table S5). However, significant differences in THSG and EG contents were detected between these groups. Combined with the trait identification and HCA-based grouping (Figure 4), these findings confirmed that Groups B and D were distinct. Therefore, the observed overlap did not undermine the classification but rather highlighted the complexity of the relationships among morphological traits, chemical composition, and hepatotoxicity in PM. Overall, the PCA score plot confirmed the classification trend based on compound content across the 61 samples, consistent with the heatmap analysis results.

### 3.3 HepaRG cell cytotoxicity results

**FIGURE 7:**
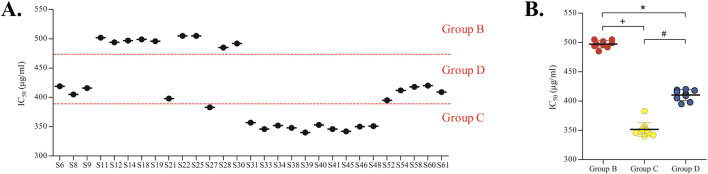
Hepatotoxicity assessment of PM samples in HepaRG cells: **(A)** IC_50_ values of different groups of commercial PM samples in HepaRG cells. **(B)** Significant differences in IC_50_ values among Groups B, C, and D (*^+#^ *p* < 0.05).

To investigate how the chemical composition of PM influences its cytotoxicity to HepaRG cells and to evaluate potential differences in toxicity among different groups of PM samples, the CCK-8 assay was employed to measure the viability of HepaRG cells across the four sample groups. IC_50_ values were determined after exposing HepaRG cells to 1–1000 μg/mL PM for 72 h (Supplementary Table S6). The positive control, APAP, showed an IC_50_ of 8.2 ± 0.9 mM, consistent with previous reports on APAP-induced toxicity in HepaRG cells (Van den Eede et al., 2015; Yokoyama et al., 2018; Deng et al., 2024). These results confirmed the sensitivity of the assay to hepatotoxicants and supported the observed differences in toxicity across PM groups. The calculated IC_50_ values aligned with the previously established PM groupings (Figure 7A). Group B samples exhibited IC_50_ values greater than 450 μg/mL, representing the lowest toxicity; Group C samples demonstrated IC_50_ values below 390 μg/mL, indicating the highest toxicity; and Group D samples had intermediate values between 390 and 450 μg/mL, reflecting moderate toxicity (Luo et al., 2024; Li et al., 2024). Significant differences in toxicity were observed among Groups B, C, and D (Figure 7B). Unexpectedly, Group A samples, despite their non-compliant THSG content, exhibited low cytotoxicity, with IC_50_ values around 540 μg/mL This finding suggests THSG and related compounds as predictive markers of hepatotoxicity.

To validate the representativeness of the 31 tested batches, THSG content, monitored as a key index in the Chinese Pharmacopeia (Committee, 2020), was compared between tested and untested batches. No significant differences were observed within the same group (*p* > 0.05, *t*-test). For example, the average THSG content in tested Group B batches was 12.54 mg/g, compared to 11.98 mg/g in untested batches (*p* = 0.32). Overall, these results demonstrated that the cytotoxic effects on HepaRG cells were closely related to the chemical composition of PM samples, providing a basis for screening potential hepatotoxic constituents.

### 3.4 Chromaticity analysis of different PM groups

**FIGURE 8:**
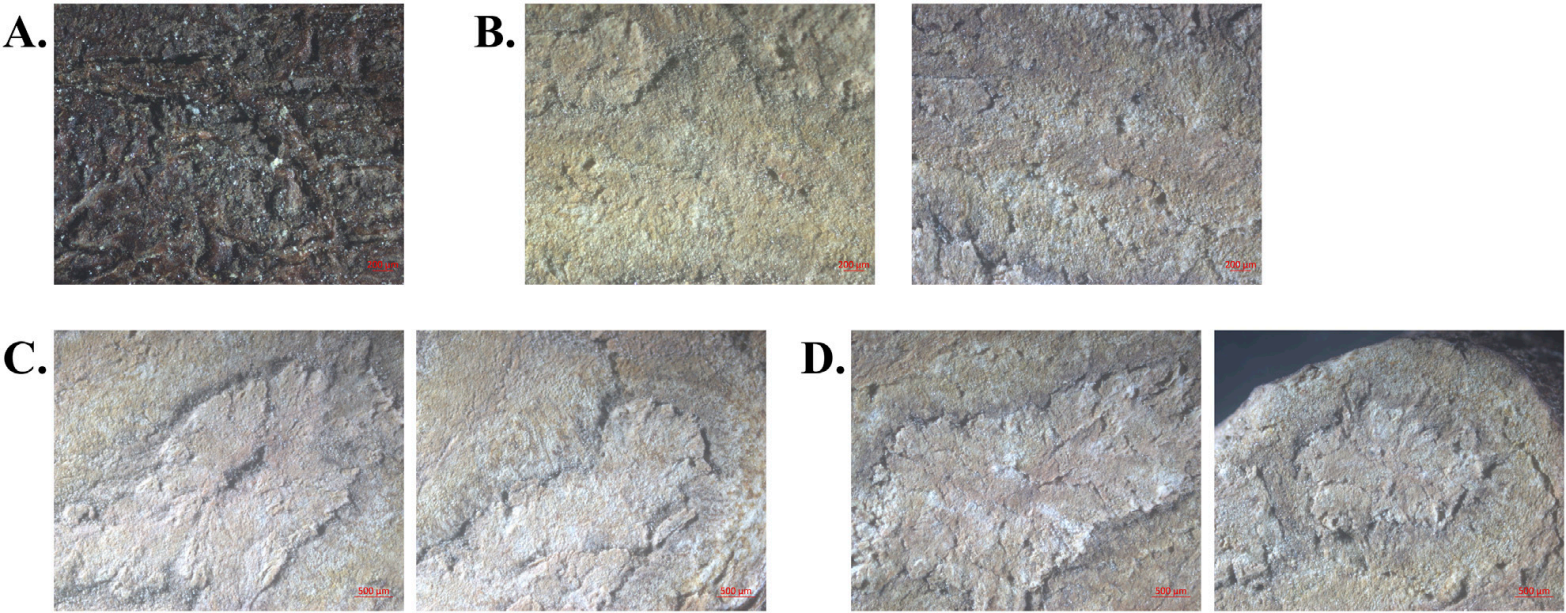
Stereoscopic microscope images of Sample S9: **(A)** The cork site. **(B)** Front and back of the phloem. **(C)** Front and back of the central xylem. **(D)** Front and back of heteromorphic vascular bundles.

**FIGURE 9:**
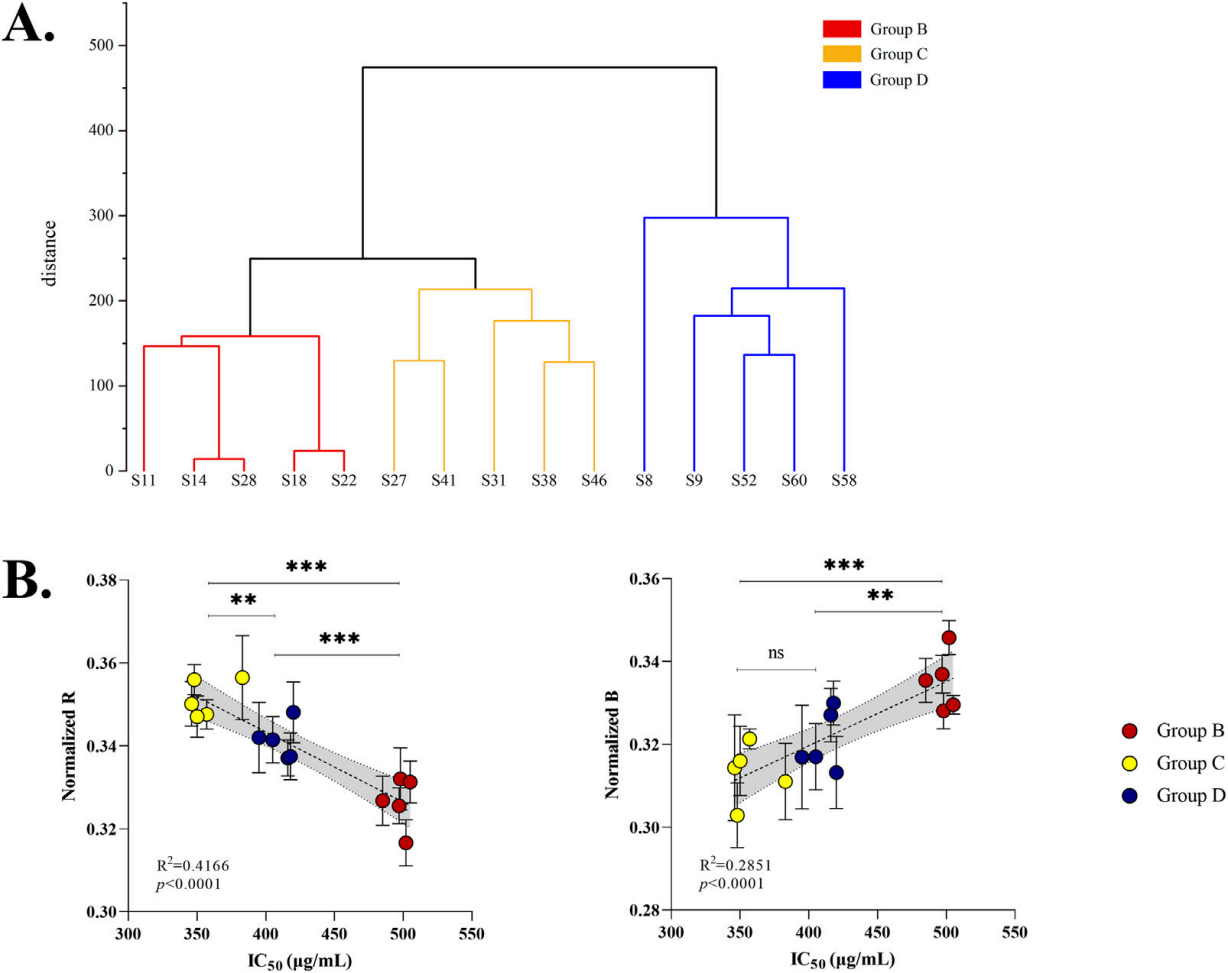
Formal statistic on the correlation between the chromaticity value and hepatotoxicity of three groups of commercial PM samples: **(A)** HCA analysis. **(B)** Regression analysis.

To ensure the accuracy and representativeness of the stereo fluorescence microscopy images, specific samples were selected: S11, S14, S18, S22, and S28 from Group B; S27, S31, S38, S41, and S46 from Group C; and S8, S9, S52, S58, and S60 from Group D. Images of the cork site (16x magnification), phloem (16x), heteromorphic vascular bundles (12x), and central xylem (12x) were collected under standardized conditions, with an identical imaging angle and light intensity (Light = 68) (Figure 8). Each sample was measured at four anatomical regions, with five sites per region on two sides, resulting in 40 chromaticity measurements per sample for mean calculation (Supplementary Table S7). Stereo fluorescence microscopy was employed to enable high-resolution, localized measurements of heterogeneous tissue structures, which bulk colorimetry could not resolve. All imaging parameters (light intensity, magnification, and angle) were rigorously standardized to ensure reproducibility. To evaluate whether chromaticity values could discriminate among PM groups, HCA was performed based on these chromaticity measurements. Clustering based on chromaticity values effectively distinguished the three groups (Figure 9A). RGB analysis revealed that Group B samples exhibited high R, G, and B values, appearing white. Group C and Group D samples showed elevated R and G values, appearing yellow. Combined with Lab values (L* for luminance, b* for yellow–blue, and a* for red–green), the color traits were identified as follows: Group B (low toxicity) appeared white, Group C (high toxicity) appeared light yellow, and Group D (medium toxicity) appeared yellow–brown. These findings indicate that chromaticity profiles, when analyzed by HCA, are associated with hepatocytotoxicity and may serve as predictors of PM toxicity. We used regression analysis to verify the correlation between chromaticity values and hepatotoxicity, Figure 9B. The results demonstrated a significant positive correlation between normalized B values and IC_50_ (p < 0.0001), and a significant negative correlation between normalized R values and IC_50_ (p < 0.0001). These findings support the reliability of using chromaticity values to predict the hepatotoxicity of PM.

It is important to note that factors such as storage conditions, drying methods, and geographic origin may influence the color stability of PM decoction pieces (Zhang et al., 2023). To minimize such variability, all samples in this study were stored under consistent conditions (room temperature, dry, and dark) prior to analysis. Furthermore, because drying methods (such as sun drying vs oven drying) and geographical origins may vary among commercially available PM, 61 batches were randomly selected to represent market diversity, thereby enhancing the representativeness and robustness of the experiment.

### 3.5 Identification of hepatotoxic constituents in different PM groups

**FIGURE 10:**
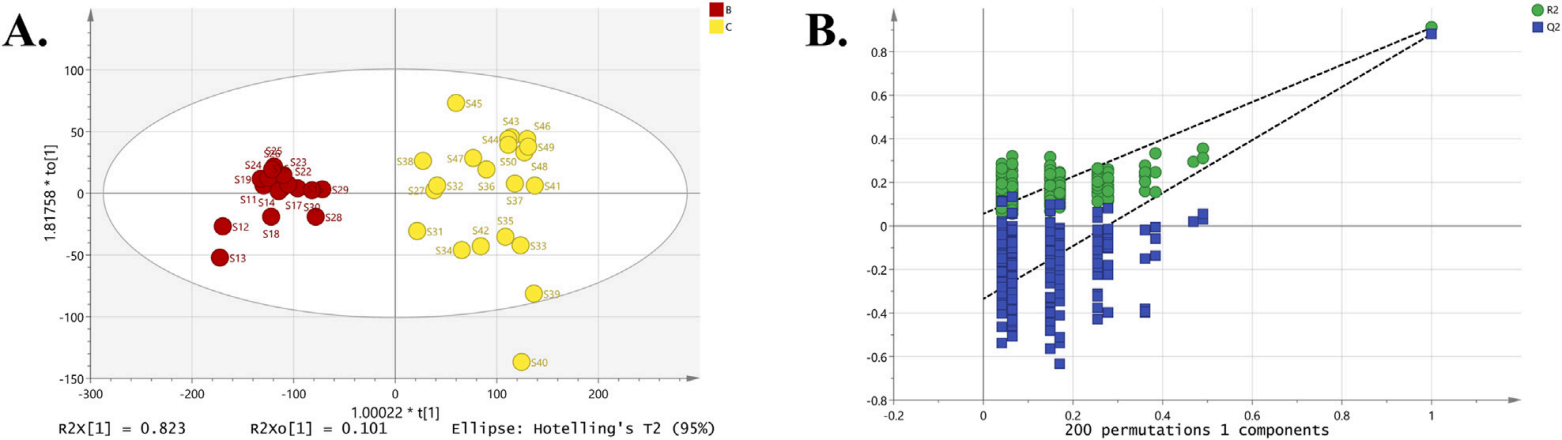
OPLS-DA analysis of Groups B and C: **(A)** The OPLS-DA score plot. **(B)** 200× permutation test for cross-validation.

**FIGURE 11:**
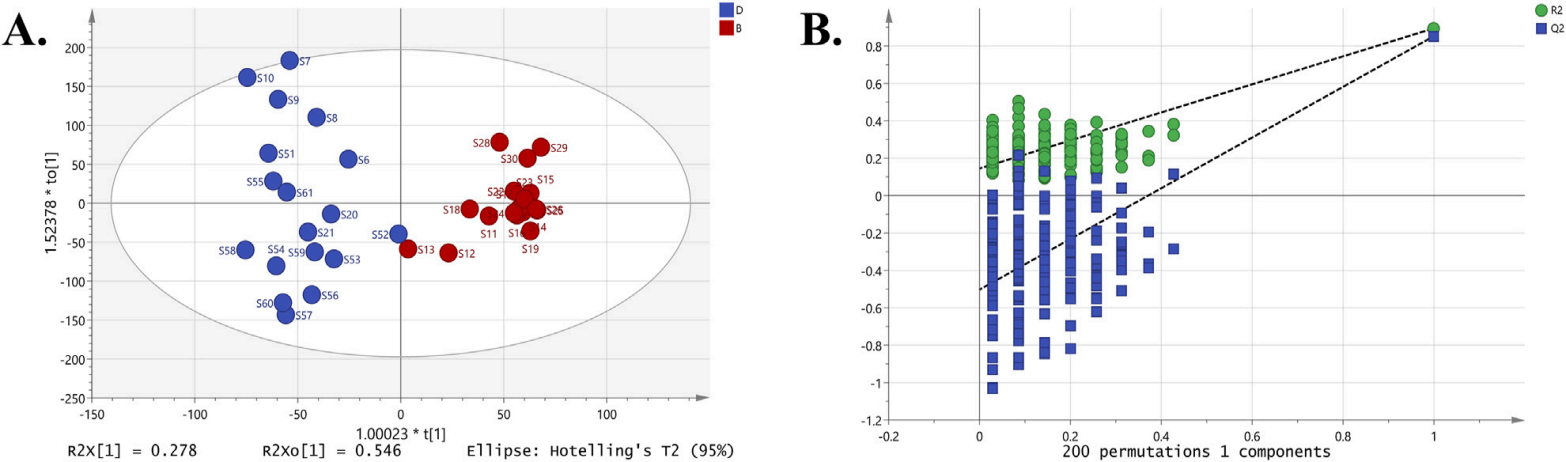
OPLS-DA analysis of Groups B and D: **(A)** The OPLS-DA score plot. **(B)** 200× permutation test for cross-validation.

Survival experiments using HepaRG cells revealed significant cytotoxicity differences among the three groups of commercial PM samples, with toxicity ranked as Group B < Group D < Group C. To minimize the influence of irrelevant factors (such as intragroup variance) and to enhance intergroup discrimination, OPLS-DA analysis was employed to identify differential toxic compounds (Yin et al., 2024; Xue et al., 2022). The first OPLS-DA analysis compared the least toxic Group B and the most toxic Group C (Figure 10). The results showed R^2^X = 92.40%, R^2^Y = 91.10%, and Q^2^ = 88.10%, indicating that the model had strong discriminative and predictive performance for distinguishing Group B from Group C. Cross-validation results from the 200× permutation test confirmed that the model was not overfitted. A second OPLS-DA analysis compared Group B with the moderately toxic Group D (Figure 11). The model achieved R^2^X = 87.30%, R^2^Y = 89.50%, and Q^2^ = 85.30%, suggesting that the expected differentiation effect was achieved, and the permutation test again confirmed that the model did not overfit. The results indicated that between Groups B and C, THSG (VIP = 4.7704) and EG (VIP = 1.0427) were potential cytotoxic markers. Between Groups B and D, THSG (VIP = 3.8540), EG (VIP = 1.8470), emodin (VIP = 1.7722), and catechin (VIP = 1.3553) were identified as differential compounds distinguishing weakly toxic and moderately toxic samples (Table 1).

**TABLE 1:** Differential chemical constituents in different groups of commercial PM samples with varying toxicity levels.

Compound no.	Group B and group C		Groups B and group D	
	VIP value	p-value	VIP value	p-value
1	0.3304	0.0143	1.7722	0.0000
2	0.1063	0.2207	0.7571	0.0002
3	0.0097	0.0000	0.0328	0.0425
4	0.0550	0.0000	0.2002	0.0000
5	1.0427	0.0000	1.8470	0.0005
6	0.0304	0.2985	0.3574	0.0367
7	0.2047	0.0000	0.3772	0.0011
8	0.0331	0.0075	0.2892	0.0000
9	0.3786	0.0004	0.7416	0.0052
10	0.0608	0.1257	0.0724	0.3682
11	0.1758	0.0200	0.3638	0.0379
12	0.0613	0.0206	0.1078	0.0769
13	4.7704	0.0000	3.8540	0.0064
14	0.4795	0.0000	0.1816	0.5677
15	0.3518	0.0000	0.0431	0.9238
16	0.2292	0.0077	0.3660	0.0002
17	0.3504	0.0004	0.8796	0.0000
18	0.9975	0.0000	1.3553	0.0000
19	0.2771	0.0001	0.1374	0.2978
20	0.0108	0.0419	0.0012	0.4211
21	0.0112	0.0000	0.0384	0.0000
22	0.2161	0.0000	0.2013	0.0625
23	0.0286	0.0029	0.1036	0.0000
24	0.0611	0.0010	0.1577	0.0002
25	0.0548	0.0000	0.0474	0.0419
26	0.3894	0.0000	0.2214	0.0732

To further validate the differential markers, a *t*-test model was established to identify compounds primarily responsible for cytotoxicity differences. When Group B was compared with Groups C and D, compounds with *p*-values <0.05 were considered as differential markers of toxicity. These results, together with VIP values >1, allowed the selection of appropriate markers (Table 1). Based on the *t*-test and VIP results, two common toxicity-related compounds (Compounds 5 and 13) were identified. THSG, widely distributed in PM samples (VIP = 4.11, *p* < 0.01), has been reported to induce hepatotoxicity under certain concentrations or ratios (Qiu et al., 2013; Xue et al., 2020). Similarly, EG (VIP = 1.51, *p* < 0.01), the main active ingredient of PM, has been shown to exert hepatotoxic effects (Zhang et al., 2020). The significance of THSG and EG as primary hepatotoxic candidates was further confirmed as they remained statistically significant (*q* < 0.05) after FDR correction for multiple testing, underscoring the robustness of these findings. In addition, previous studies have shown that when stilbene glycosides are combined with anthraquinones, they induce greater metabolic pathway disruption compared to anthraquinones alone, suggesting synergistic toxic interactions between these compound classes (Zhang et al., 2020; Lin et al., 2015).

### 3.6 Combined toxicity of potential hepatotoxic constituents in PM

**FIGURE 12:**
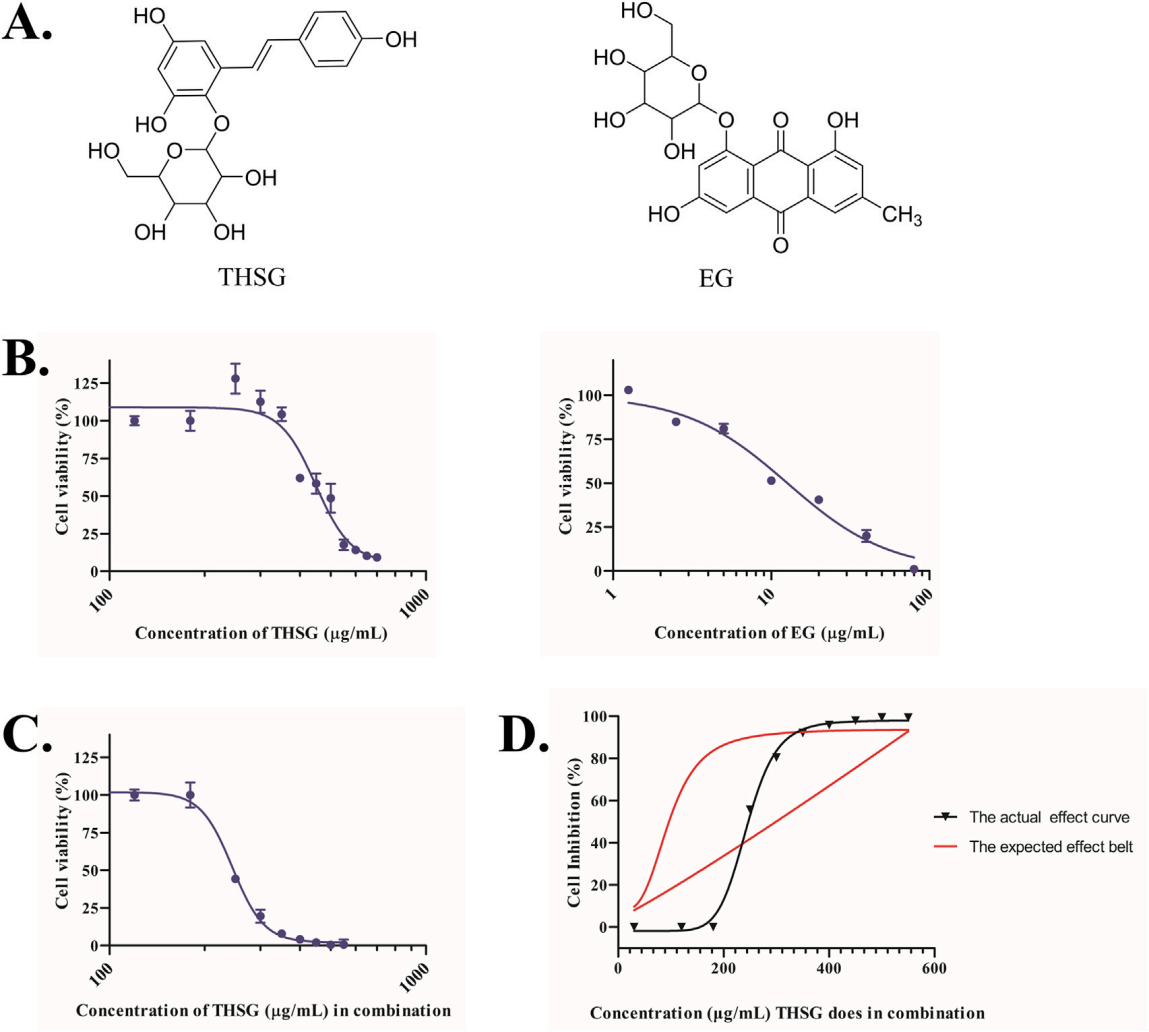
Synergistic activity of THSG and EG: **(A)** Chemical structures of THSG and EG. **(B)** CCK-8 assay–based determination of cell viability after 72 h of THSG or EG treatment. **(C)** CCK-8 assay–based determination of cell viability after co-administration of THSG and EG at different doses. **(D)** The dose–effect belt comparing predicted and observed dose–effect curves for THSG + EG.

The structures of THSG and E.G., are shown in Figure 12A. Their cytotoxic effects on HepaRG hepatocytes were evaluated individually at concentrations of 30–700 μg/mL for THSG and 1–80 μg/mL for EG. THSG was non-toxic at concentrations up to 350 μg/mL; however, at 400–500 μg/mL, only 50% of the cells survived (Figure 12B). In contrast, E.G., exhibited stronger toxicity, with 50% inhibition observed at 10 μg/mL, and complete cell death was visually observed at 80 μg/mL. Mass spectrometry confirmed the presence of both compounds in all PM samples, although their relative levels varied across Groups B, C, and D, corresponding to different degrees of hepatocytotoxicity. These findings suggest that the ratio of THSG to E.G., is an important determinant of toxicity.

To further investigate the antiproliferative effects of THSG and EG, 10 concentrations of THSG (30, 120, 180, 250, 300, 350, 400, 450, 500, and 550 μg/mL) were combined with 10 concentrations of E.G., (1.25, 2.5, 5, 7.5, 10, 12.5, 15, 17.5, 20, and 22.5 μg/mL). These concentration ranges not only covered the IC_50_ values of the two compounds but also reflected physiologically achievable concentrations *in vivo*. As reported by Yu et al. (2017), THSG can accumulate in the liver at concentrations up to several hundred µg/mL, which is consistent with the mid-to-high concentration range tested in this study (250–550 μg/mL). After 72 h of incubation, the IC_50_ values for THSG and EG were 417.0 μg/mL and 12.7 μg/mL, respectively. When the two compounds were co-incubated, the IC_50_ decreased to 247.7 + 7.5 μg/mL (Figure 12C), indicating that the toxicity of EG was significantly enhanced when THSG was co-administered at non-toxic concentrations (250–350 μg/mL). These findings demonstrated that THSG and EG exerted greater cytotoxic effects when combined. The hepatotoxic interactions between THSG and EG (additive, synergistic, or antagonistic) were evaluated using the “One-belt, One-line” model (Yuan and Chen, 2019). In this approach, the belt represents the observed dose–effect relationship of the two-drug combination (Figure 12D), while the red curve represents the expected additive effect. When the observed efficacy falls within the expected additive range, the interaction is classified as additive; when it exceeds the range, it is synergistic; and when it is lower than expected, it is antagonistic. Our results showed that combinations of 30–180 μg/mL THSG with 1.25–5 μg/mL EG exhibited antagonistic effects. In contrast, combinations of 250–350 μg/mL THSG with 10–15 μg/mL EG showed additive effects. Notably, combinations of 400–550 μg/mL THSG with 15–22.5 μg/mL EG produced synergistic effects (Figure 12D; Table 2). The CI was used to further quantify the additive, synergistic, and antagonistic interactions between THSG and EG (Table 2). Both the “One-belt, One-line” model and CI values consistently indicated that increasing the concentrations of the two compounds enhanced hepatotoxic effects, thereby narrowing the drug safety window and increasing the risk of liver injury. The consistency of results between the approaches confirmed the classification of additional, synergistic, and antagonistic interactions between THSG and EG. Quantitative analysis revealed that THSG and EG were widely distributed in PM samples (4.90–57.24 mg/g and 0.10–4.73 mg/g, respectively). Notably, Group C samples, which exhibited the strongest cytotoxicity, contained higher levels of THSG (32.58–57.24 mg/g) and E.G., (1.45–4.73 mg/g). These elevated concentrations were positively correlated with toxicity, consistent with the interaction patterns observed in the “One-belt, One-line” model. Similar trends were observed in Group D, which displayed moderate toxicity.

**TABLE 2:** Determination of THSG–EG interactions using dose-based combination index (CI_d_) values.

Combination doses		Predictable additive effects (%)		Real effects (%)	CI_d_		Judgements
THSG (µg/mL)	E.G., (µg/mL)	f (T + T_E_)	g (E + E_T_)	Y_obs_	CI_d1_	CI_d2_	
30.00	1.25	8.03	9.62	0.00	0.0000 < 1	0.0000 < 1	Antagonistic
120.00	2.50	19.42	64.88	0.00	0.0000 < 1	0.0000 < 1	Antagonistic
180.00	5.00	33.23	83.70	0.00	0.0000 < 1	0.0000 < 1	Antagonistic
250.00	7.50	42.44	89.90	55.73	1.3132 > 1	0.6199 < 1	Additive
300.00	10.00	49.74	91.71	80.48	1.6180 > 1	0.8775 < 1	Additive
350.00	12.50	56.91	92.59	92.13	1.6189 > 1	0.9950 < 1	Additive
400.00	15.00	65.68	93.05	95.87	1.4596 > 1	1.0303 > 1	Synergistic
450.00	17.50	76.13	93.31	97.92	1.2862 > 1	1.0494 > 1	Synergistic
500.00	20.00	85.59	93.46	99.44	1.1618 > 1	1.0640 > 1	Synergistic
550.00	22.50	92.31	93.55	99.40	1.0769 > 1	1.0625 > 1	Synergistic

f(T + T_E_) and g(E + E_T_) were calculated using the “One-belt, One-line” method (Yuan and Chen, 2019), while Y_obs_ was determined by the CCK8 assay. f(T + T_E_) represents the expected additive effect of THSG combined with EG based on equivalent dose conversion when THSG, was the target drug, whereas g(E + E_T_) represents the expected additive effect of EG combined with THSG based on equivalent dose conversion when the EG was the target drug; CI_d_, was calculated as Y_obs_/f(T + T_E_) or Y_obs_/g(E + E_T_). The determination criteria were as follows: synergy, CI_d1_ > 1 and CI_d2_ > 1; addition, CI_d1_ ≤ 1 and CI_d2_ ≤ 1; and antagonism, CI_d1_ < 1 and CI_d2_ < 1.

Overall, these findings demonstrate that the hepatotoxicity of PM is not attributable to a single compound but arises from interactions among multiple constituents. The dose-dependent synergistic toxicity observed between THSG and E.G., highlights the risks associated with high-dose consumption or specific compound combinations in PM products.

## 4 Discussion and conclusion

PM, widely used as both a medicinal and edible product, has a consistently high market demand. However, in recent years, its potential hepatotoxicity has raised significant public health concerns, while the identification of its toxic constituents and their interactions remains incomplete. In this study, we established a multiparameter evaluation model integrating chemical composition, hepatotoxicity, and morphological traits to systematically assess the safety of commercial PM samples. Our findings not only confirmed the hepatotoxic potential of specific chemical constituents but also demonstrated the utility of a novel combinatorial approach for toxicity evaluation. A total of 61 batches of commercial PM samples from different regions of China were analyzed. Based on previous studies (Xiong et al., 2024; Wang et al., 2024), we characterized their compound profiles, HepaRG cytotoxicity, and chromaticity values to reveal associations among morphological traits, chemical composition, and hepatotoxic effects. Using UPLC-ESI-QqQ-MS/MS analysis in combination with HepaRG cytotoxicity assays, we first confirmed a relationship between chemical composition and hepatotoxicity. We then established that chromaticity was correlated with hepatocytotoxicity: white decoction pieces were relatively non-toxic, light-yellow pieces were highly toxic, and yellow-brown pieces showed moderate toxicity. Statistical analysis was subsequently conducted to identify potential hepatotoxic constituents. Although previous studies have investigated the toxic components of PM, most have focused on single compounds and overlooked possible synergistic effects (Hu et al., 2021; Xing et al., 2023). For the first time, by applying the “One-belt, One-line” drug combination model, we investigated the dose–toxicity relationships of two potential hepatotoxic constituents, THSG and EG, and demonstrated that their additive and synergistic hepatotoxicity were significantly enhanced at higher concentrations. The main innovations of this study are reflected in the following aspects.

First, we identified THSG and EG as the primary candidate hepatotoxic constituents of PM. Both OPLS-DA and *t*-test analyses revealed significant differences in these compounds among PM groups with varying toxicity levels (VIP >1, *p* < 0.05). Importantly, we demonstrated that non-toxic doses of THSG significantly enhanced the hepatotoxicity of EG, and their co-administration produced concentration-dependent synergistic effects, thereby narrowing the drug safety window. This underscores the importance of considering compound ratios and interactions in PM-induced liver injury, moving beyond the traditional focus on single-component toxicity. Similar findings have been reported in the literature. For example, anthraquinones such as emodin may act as reactive electrophiles that covalently bind to nucleophilic molecules such as glutathione, amino acids, and proteins, thereby inducing toxicity (Qin et al., 2016; Wang et al., 2019). They may also undergo phase I and II metabolism, with their metabolites inhibiting uridine diphosphate glucuronosyltransferase 1A1, leading to bilirubin accumulation and hepatotoxicity (Zhang et al., 2020; Huang et al., 2019). THSG, a stilbene glycoside, generally does not cause liver damage when administered alone. However, in lipopolysaccharide-induced inflammatory stress models and acetaminophen-induced hepatotoxicity models, it has been shown to exacerbate liver injury. When combined with compounds such as EG, THSG can inhibit metabolic enzymes, resulting in drug accumulation and hepatotoxicity (Zhang et al., 2020). Both THSG and emodin have been detected in the serum of patients with PM-induced liver injury (Panis et al., 2005), further supporting their clinical relevance. Our findings, therefore, support THSG and EG as key hepatotoxic constituents of PM and demonstrate that their combined administration enhances toxicity. Notably, the dose–response relationship of their combined administration had not been previously explored.

Second, we introduced the “One-belt, One-line” drug combination model as a novel methodological approach for evaluating dose–effect relationships of combined phytochemicals. While this model has been primarily applied to efficacy studies in cancer and cardiovascular diseases (Yuan and Chen, 2019; Tang et al., 2020), its principles are equally applicable to toxicological interactions, as demonstrated in recent studies using combination index methods for toxicity assessment (Wu et al., 2024; Sriramulu et al., 2024). In this study, we adapted the model to quantify the hepatotoxic interactions between THSG and EG, providing a visual and quantitative framework for assessing combined toxicity in herbal medicines. Our results revealed that at low concentrations, THSG and EG exhibited antagonistic effects; at intermediate concentrations, they produced additive effects; and at high concentrations, they showed synergistic toxicity. Notably, the combination of the two compounds at high concentrations significantly narrowed the drug safety window. Combined with quantitative data on compound concentrations, these findings suggest that variations in the doses and ratios of THSG and EG across different batches of PM samples contribute to the observed differences in hepatotoxicity. This model offers a quantitative and visual framework for assessing combined toxicity, an approach rarely applied in herbal medicine safety studies but holds great promise for advancing research on multicomponent interactions. Although this study did not experimentally validate the mechanistic basis of THSG–EG synergistic hepatotoxicity, existing literature provides plausible pathways. THSG has been reported to inhibit key drug-metabolizing enzymes such as UDP-glucuronosyltransferase and cytochrome P450 enzymes, potentially delaying the metabolism and excretion of anthraquinones such as EG, leading to their accumulation and enhanced toxicity (Zhang et al., 2020; Yu et al., 2017). Furthermore, emodin and its derivatives can induce oxidative stress through mitochondrial dysfunction and ROS generation, which may be exacerbated by THSG-mediated metabolic inhibition (Qin et al., 2016; Wang et al., 2019). We therefore hypothesize that the synergy observed at high concentrations results from a combined effect of metabolic inhibition and oxidative damage, ultimately accelerating hepatocyte apoptosis. Future studies employing metabolomics, transcriptomics, and enzyme activity assays are warranted to validate these mechanisms.

Third, this study provides practical implications for the safety monitoring of PM and other herbal medicines. We established a correlation between the color traits of PM decoction pieces and their hepatotoxicity: white pieces were associated with lower toxicity, while yellow to brown pieces indicated higher risk. This suggests that simple, non-destructive color assessment could serve as a preliminary screening tool for PM quality control. Furthermore, the multi-index model integrating chemical, toxicological, and morphological trait data offers a comprehensive strategy for evaluating the safety of complex herbal products, supporting improved regulatory oversight and consumer protection. This approach aligns with recently proposed guidelines for the safe use of PM, which emphasize the importance of chemical standardization and toxicity monitoring (Xiao et al., 2024). In addition to identifying toxic constituents and their interactions, exploring detoxification strategies is also essential. For example, Xiu et al. (2024) demonstrated that *Paeoniae Radix Alba* alleviates PM-induced liver injury by modulating macrophage polarization toward the M2 phenotype, which is associated with anti-inflammatory and tissue-repair functions. These findings suggest that combining PM with hepatoprotective agents such as *Paeoniae Radix Alba* may enhance its safety profile while preserving its therapeutic benefits. Future studies should investigate such combinatorial approaches to mitigate the hepatotoxic risks associated with PM consumption.

In conclusion, this study identifies THSG and EG as key hepatotoxic constituents of PM and demonstrates their synergistic toxicity using a novel combinatorial model. By integrating chemical analysis, cytotoxicity assays, and morphological trait characterization, we established a robust framework for evaluating the safety of herbal medicines. Although preliminary experiments compared multiple cell lines, including L-02, HepG2, and PRLs, the differentiated HepaRG cell line was ultimately selected as the primary model. HepaRG cells are well characterized, with stable expression of key phase I/II drug-metabolizing enzymes and liver-specific functions (Aninat et al., 2006; Di Cocco et al., 2019). Thus, they provide a reliable platform for high-throughput hepatotoxicity screening and are particularly suitable for investigating metabolism-dependent toxicity, although they remain an *in vitro* simplification of the human liver (Stanley and Wolf, 2022; De Jong et al., 2024). Future studies should therefore validate these interactions in more physiologically complex models, such as 3D hepatocyte spheroids or *in vivo* systems. These advanced models are expected to better account for systemic factors, immune responses, and multi-tissue interactions that may influence the hepatotoxic potential of PM. Confirming the synergistic effects of THSG and EG in these models will significantly enhance the translational relevance of our findings and provide a more comprehensive safety assessment of PM.

## Data Availability

The original contributions presented in the study are included in the article/Supplementary Material, further inquiries can be directed to the corresponding author.
