# Supplementary material for: Integrated chemometric evaluation of morphological traits, chemical composition, and hepatotoxicity of Polygonum multiflorum
Source: Front Pharmacol. 2025 Oct 23;16:1666300. doi: 10.3389/fphar.2025.1666300 (PMC12588903; doi:10.3389/fphar.2025.1666300)
Supplement: Supplementary file 1 [file Supplementaryfile1.docx]

Supplementary Material

# Supplementary Tables

**Table S1.** Reference substance information for quantitative analysis of PM.

| Compound No. | Compound name | CAS number | Ipurity (%) | Source |
| --- | --- | --- | --- | --- |
| 1 | Emodin | 518-82-1 | 96 | NIFDC |
| 2 | Physcion | 521-61-9 | 99 | NIFDC |
| 3 | Rhein | 478-43-3 | 98 | NIFDC |
| 4 | 1-Methyl emodin | 3775-08-4 | 98 | Nature Standard |
| 5 | Emodin-8-glucoside | 23313-21-5 | 98 | Preferred |
| 6 | Physcion-8-glucoside | 23451-01-6 | 98 | Nature Standard |
| 7 | Emodin-1-glucoside | 38840-23-2 | 98 | Preferred |
| 8 | Emodin-6-glucoside | 34298-85-6 | 95 | Nature Standard |
| 9 | Emodin-8-*O*-(6'-methylmalonyl)-glucopyranoside | 928262-58-2 | 98 | Nature Standard |
| 10 | Physcion-8-*O*-(6'-methylmalonyl)-glucopyranoside | 1345826-33-6 | 98 | Nature Standard |
| 11 | *Trans*-emodin dianthrones | 61281-20-7 | 98 | Nature Standard |
| 12 | *Cis*-emodin dianthrones | 61281-19-4 | 98 | Nature Standard |
| 13 | 2,3,5,4'-Tetrahydroxystilbene-2-*O-*β*-D*-glucoyranoside | 82373-94-2 | 91 | NIFDC |
| 14 | *Cis*-2,3,5,4'-tetrahydroxystilbene-2-*O-*β*-D*-glucoyranoside | / | 98 | Preferred |
| 15 | 2,3,5,4'-Tetrahydroxystilbene-2-*O*-(2”-*O*-feruloyl)-β*-D*-glucopyranoside | 359406-98-7 | 98 | Nature Standard |
| 16 | Polydatin | 27208-80-6 | 87.3 | NIFDC |
| 17 | Resveratrol | 501-36-0 | 99 | NIFDC |
| 18 | Catechin | 18829-70-4 | 95.1 | NIFDC |
| 19 | Epicatechin | 490-46-0 | 99 | NIFDC |
| 20 | Hyperoside | 482-36-0 | 94.9 | NIFDC |
| 21 | Rutin | 153-18-4 | 91.7 | NIFDC |
| 22 | Gallic acid | 149-91-7 | 90.1 | NIFDC |
| 23 | *p*-Hydroxybenzoic acid | 99-96-7 | 99 | Nature Standard |
| 24 | *p*-Hydroxybenzaldehyde | 123-08-0 | 99 | Nature Standard |
| 25 | *p*-Coumaric acid | 501-98-4 | 98 | Nature Standard |
| 26 | Torachrysone 8-*O*-glucoside | 64032-49-1 | 98 | Nature Standard |

**Table S2.** The detailed information of the PM samples used in this work.

| Sample No. | Growing areas | Sample No. | Growing areas |
| --- | --- | --- | --- |
| S1 | Honghe, Yunnan | S32 | Kaili, Guizhou |
| S2 | Honghe, Yunnan | S33 | Kaili, Guizhou |
| S3 | Honghe, Yunnan | S34 | Kaili, Guizhou |
| S4 | Honghe, Yunnan | S35 | Kaili, Guizhou |
| S5 | Honghe, Yunnan | S36 | Bijie, Guizhou |
| S6 | Qujing, Yunnan | S37 | Bijie, Guizhou |
| S7 | Qujing, Yunnan | S38 | Bijie, Guizhou |
| S8 | Qujing, Yunnan | S39 | Bijie, Guizhou |
| S9 | Qujing, Yunnan | S40 | Bijie, Guizhou |
| S10 | Qujing, Yunnan | S41 | Anshun, Guizhou |
| S11 | Qujing, Yunnan | S42 | Anshun, Guizhou |
| S12 | Bozhou, Anhui | S43 | Anshun, Guizhou |
| S13 | Bozhou, Anhui | S44 | Anshun, Guizhou |
| S14 | Bozhou, Anhui | S45 | Anshun, Guizhou |
| S15 | Bozhou, Anhui | S46 | Tongren, Guizhou |
| S16 | Bozhou, Anhui | S47 | Tongren, Guizhou |
| S17 | Fuyang, Anhui | S48 | Tongren, Guizhou |
| S18 | Fuyang, Anhui | S49 | Tongren, Guizhou |
| S19 | Fuyang, Anhui | S50 | Tongren, Guizhou |
| S20 | Fuyang, Anhui | S51 | Dazhou, Sichuan |
| S21 | Fuyang, Anhui | S52 | Dazhou, Sichuan |
| S22 | Gaozhou, Guangdong | S53 | Dazhou, Sichuan |
| S23 | Gaozhou, Guangdong | S54 | Dazhou, Sichuan |
| S24 | Gaozhou, Guangdong | S55 | Dazhou, Sichuan |
| S25 | Gaozhou, Guangdong | S56 | Yibin, Sichuan |
| S26 | Gaozhou, Guangdong | S57 | Yibin, Sichuan |
| S27 | Deqing, Guangdong | S58 | Yibin, Sichuan |
| S28 | Deqing, Guangdong | S59 | Yibin, Sichuan |
| S29 | Deqing, Guangdong | S60 | Yibin, Sichuan |
| S30 | Deqing, Guangdong | S61 | Deqing, Guangdong |
| S31 | Kaili, Guizhou | - | - |

**Table S3.** Concentrations of anthraquinones in PM (µg/g)

| Sample | Emodin | Physcion | Rhein | 1-Methyl emodin | Emodin-8-glucoside | Physcion-8-glucoside | Emodin-1-glucoside | Emodin-6-glucoside | Emodin-8-*O*-(6'-methylmalonyl)-glucopyranoside | Physcion-8-*O*-(6'-methylmalonyl)-glucopyranoside | Trans-emodin dianthrones | Cis-emodin dianthrones |
| --- | --- | --- | --- | --- | --- | --- | --- | --- | --- | --- | --- | --- |
| S1 | 60.30 | 81.14 | 0.08 | 1.86 | 312.15 | 52.56 | 22.67 | 1.09 | 249.88 | 23.15 | 1.47 | 0.18 |
| S2 | 1.48 | 53.96 | 0.03 | 0.91 | 101.80 | 8.86 | 10.42 | 0.20 | 33.77 | 5.76 | 1.11 | 0.16 |
| S3 | 41.18 | 69.96 | 0.10 | 2.36 | 197.25 | 31.32 | 17.82 | 0.61 | 164.38 | 21.59 | 1.24 | 0.16 |
| S4 | 71.82 | 86.37 | 0.08 | 10.03 | 208.71 | 33.62 | 18.53 | 1.01 | 155.15 | 17.42 | 0.96 | 0.14 |
| S5 | 33.76 | 75.05 | 0.52 | 18.85 | 162.05 | 34.82 | 18.50 | 1.68 | 128.81 | 24.68 | 1.11 | 0.15 |
| S6 | 702.34 | 404.89 | 0.32 | 6.10 | 3387.85 | 385.52 | 222.24 | 28.33 | 620.38 | 98.09 | 17.04 | 1.72 |
| S7 | 1040.72 | 406.08 | 0.34 | 3.19 | 3743.79 | 344.84 | 185.13 | 34.12 | 642.98 | 80.13 | 9.89 | 0.81 |
| S8 | 987.88 | 411.81 | 0.36 | 8.98 | 3386.41 | 322.85 | 194.77 | 26.44 | 780.26 | 83.59 | 9.90 | 0.85 |
| S9 | 1458.11 | 426.19 | 0.56 | 16.03 | 4236.49 | 289.15 | 195.17 | 27.66 | 1067.09 | 61.20 | 18.31 | 1.69 |
| S10 | 1606.15 | 413.95 | 0.86 | 26.52 | 4444.78 | 330.74 | 191.71 | 37.32 | 777.29 | 75.58 | 11.08 | 0.98 |
| S11 | 631.51 | 262.07 | 0.00 | 0.04 | 406.07 | 82.20 | 21.79 | 12.22 | 216.58 | 75.57 | 141.01 | 16.14 |
| S12 | 1006.43 | 248.81 | 0.00 | 0.20 | 1205.22 | 170.37 | 78.32 | 14.85 | 497.95 | 49.05 | 3.42 | 0.40 |
| S13 | 1276.43 | 264.09 | 0.00 | 0.25 | 1623.17 | 197.47 | 94.63 | 18.36 | 616.30 | 42.02 | 6.83 | 0.75 |
| S14 | 471.67 | 284.70 | 0.00 | 0.08 | 1764.75 | 290.36 | 132.32 | 17.38 | 790.35 | 63.00 | 3.09 | 0.37 |
| S15 | 509.30 | 262.07 | 0.00 | 0.08 | 1661.33 | 214.42 | 110.84 | 13.61 | 677.71 | 63.27 | 2.99 | 0.36 |
| S16 | 537.35 | 279.56 | 0.00 | 0.10 | 1523.31 | 222.36 | 99.80 | 14.35 | 763.03 | 70.03 | 4.13 | 0.42 |
| S17 | 403.90 | 266.26 | 0.00 | 0.08 | 1558.23 | 176.66 | 117.74 | 9.79 | 686.79 | 65.79 | 2.55 | 0.27 |
| S18 | 472.50 | 290.67 | 0.00 | 0.05 | 1723.68 | 199.50 | 128.17 | 11.60 | 644.74 | 50.09 | 2.95 | 0.30 |
| S19 | 437.30 | 297.58 | 0.00 | 0.06 | 1311.96 | 199.63 | 104.01 | 9.75 | 579.64 | 65.24 | 2.41 | 0.25 |
| S20 | 2171.05 | 695.70 | 0.00 | 0.27 | 852.39 | 118.08 | 60.31 | 19.74 | 1222.39 | 48.22 | 5.45 | 0.47 |
| S21 | 2382.30 | 721.98 | 0.00 | 0.40 | 817.88 | 108.02 | 52.66 | 20.15 | 1214.43 | 36.21 | 4.31 | 0.39 |
| S22 | 964.54 | 289.93 | 0.00 | 0.07 | 438.67 | 171.44 | 25.41 | 13.67 | 411.97 | 100.86 | 293.74 | 39.96 |
| S23 | 798.67 | 248.69 | 0.00 | 0.09 | 486.96 | 200.30 | 25.20 | 14.20 | 403.99 | 96.27 | 255.73 | 32.32 |
| S24 | 794.15 | 246.65 | 0.00 | 0.13 | 371.14 | 123.78 | 20.17 | 7.95 | 393.54 | 103.16 | 267.82 | 32.02 |
| S25 | 707.17 | 234.01 | 0.00 | 0.15 | 379.35 | 140.05 | 23.15 | 9.70 | 328.71 | 81.14 | 214.53 | 25.72 |
| S26 | 737.56 | 234.23 | 0.00 | 0.11 | 332.33 | 122.69 | 20.09 | 8.69 | 318.87 | 91.60 | 280.61 | 32.06 |
| S27 | 525.47 | 187.92 | 0.00 | 0.02 | 1649.32 | 325.15 | 157.52 | 39.37 | 213.31 | 76.67 | 1.41 | 0.22 |
| S28 | 557.43 | 315.94 | 0.00 | 0.00 | 1012.04 | 39.47 | 33.55 | 9.61 | 137.51 | 103.75 | 16.36 | 1.96 |
| S29 | 397.43 | 321.09 | 0.00 | 0.00 | 1089.66 | 59.37 | 36.48 | 6.97 | 133.81 | 88.92 | 9.06 | 1.06 |
| S30 | 241.80 | 320.82 | 0.00 | 0.00 | 1471.79 | 79.32 | 45.85 | 15.01 | 130.16 | 110.76 | 4.98 | 0.57 |
| S31 | 517.24 | 287.55 | 0.28 | 9.23 | 2882.03 | 140.85 | 139.00 | 13.48 | 946.27 | 71.90 | 8.25 | 0.81 |
| S32 | 430.26 | 300.09 | 0.29 | 9.85 | 2419.30 | 176.75 | 158.07 | 17.52 | 550.19 | 69.64 | 4.47 | 0.45 |
| S33 | 531.21 | 349.92 | 0.32 | 13.23 | 3983.00 | 233.71 | 218.62 | 22.27 | 882.63 | 67.00 | 6.54 | 0.65 |
| S34 | 509.93 | 390.71 | 0.26 | 7.60 | 3432.78 | 209.75 | 188.90 | 24.95 | 1162.39 | 97.55 | 8.60 | 0.82 |
| S35 | 467.72 | 372.90 | 0.21 | 6.62 | 3040.71 | 197.78 | 192.89 | 19.05 | 871.91 | 64.20 | 9.05 | 0.96 |
| S36 | 382.92 | 297.22 | 0.04 | 1.48 | 2161.09 | 264.00 | 136.16 | 16.59 | 613.41 | 130.77 | 6.03 | 0.61 |
| S37 | 401.34 | 288.82 | 0.04 | 1.18 | 2236.96 | 242.59 | 129.85 | 17.50 | 648.05 | 105.80 | 2.11 | 0.23 |
| S38 | 419.76 | 280.42 | 0.31 | 8.55 | 2312.83 | 221.18 | 123.54 | 18.41 | 682.70 | 80.83 | 3.25 | 0.34 |
| S39 | 870.93 | 584.99 | 0.24 | 6.76 | 3501.87 | 176.35 | 165.99 | 16.83 | 766.39 | 61.71 | 16.89 | 1.77 |
| S40 | 652.23 | 522.33 | 0.18 | 4.58 | 4726.71 | 147.34 | 198.61 | 15.25 | 962.27 | 71.99 | 14.84 | 1.60 |
| S41 | 541.67 | 285.42 | 0.06 | 4.28 | 2549.48 | 169.50 | 130.37 | 14.47 | 1006.61 | 88.74 | 26.94 | 3.38 |
| S42 | 517.82 | 432.15 | 0.34 | 10.60 | 3423.00 | 127.17 | 166.25 | 16.75 | 840.88 | 61.06 | 17.13 | 1.93 |
| S43 | 326.36 | 229.32 | 0.16 | 8.38 | 1988.48 | 168.63 | 89.99 | 20.37 | 853.69 | 82.70 | 12.16 | 1.36 |
| S44 | 334.28 | 222.65 | 0.04 | 1.75 | 1906.40 | 178.80 | 99.65 | 12.06 | 682.58 | 92.63 | 9.92 | 1.12 |
| S45 | 358.07 | 209.61 | 0.00 | 1.86 | 1446.64 | 125.09 | 94.57 | 9.71 | 682.71 | 93.51 | 18.25 | 2.10 |
| S46 | 357.05 | 251.62 | 0.07 | 1.73 | 1780.84 | 53.94 | 87.61 | 9.37 | 589.47 | 50.52 | 22.82 | 2.78 |
| S47 | 417.64 | 225.27 | 0.10 | 1.94 | 1795.24 | 117.21 | 103.19 | 11.03 | 613.20 | 104.88 | 32.13 | 4.01 |
| S48 | 369.65 | 247.46 | 0.29 | 7.69 | 2107.72 | 148.82 | 106.15 | 12.69 | 670.98 | 124.26 | 18.41 | 2.19 |
| S49 | 409.96 | 222.86 | 0.07 | 1.47 | 2081.53 | 163.30 | 101.15 | 10.95 | 679.28 | 105.11 | 30.54 | 3.71 |
| S50 | 265.63 | 209.24 | 0.08 | 1.12 | 1904.73 | 189.84 | 96.48 | 16.49 | 480.69 | 105.31 | 4.80 | 0.56 |
| S51 | 1519.46 | 321.87 | 0.32 | 10.56 | 1658.40 | 268.75 | 84.18 | 53.00 | 312.52 | 93.30 | 25.16 | 1.76 |
| S52 | 903.67 | 285.79 | 0.27 | 11.62 | 1651.37 | 148.43 | 128.52 | 16.73 | 483.79 | 32.81 | 10.78 | 1.21 |
| S53 | 1211.57 | 541.91 | 0.48 | 15.82 | 1654.89 | 119.95 | 106.35 | 25.20 | 398.16 | 42.76 | 38.26 | 4.52 |
| S54 | 1279.13 | 788.57 | 0.53 | 30.31 | 1606.51 | 151.60 | 53.98 | 45.38 | 391.00 | 67.84 | 21.13 | 14.79 |
| S55 | 1038.79 | 229.94 | 0.16 | 3.09 | 1554.62 | 195.98 | 65.50 | 41.22 | 469.47 | 86.74 | 17.10 | 3.74 |
| S56 | 1619.64 | 616.04 | 0.31 | 16.29 | 1612.08 | 155.74 | 114.13 | 32.76 | 825.54 | 62.66 | 48.21 | 6.78 |
| S57 | 1467.49 | 318.26 | 0.33 | 9.52 | 1583.35 | 158.49 | 69.13 | 36.99 | 408.43 | 74.70 | 32.66 | 5.26 |
| S58 | 1953.38 | 840.72 | 0.33 | 16.42 | 2233.19 | 173.02 | 132.92 | 34.51 | 1002.20 | 67.82 | 41.85 | 5.23 |
| S59 | 1704.16 | 344.02 | 0.37 | 18.17 | 1915.66 | 93.20 | 119.36 | 23.37 | 917.28 | 47.23 | 39.89 | 5.15 |
| S60 | 2055.05 | 574.39 | 5.30 | 9.56 | 1720.47 | 215.70 | 114.95 | 28.94 | 644.21 | 57.52 | 40.87 | 12.24 |
| S61 | 1244.80 | 321.17 | 0.20 | 12.86 | 2647.26 | 461.41 | 197.40 | 36.17 | 663.45 | 146.94 | 9.04 | 1.01 |

**Table S4**

Concentrations of stilbene glycosides in PM (µg/g)

| Sample | 2,3,5,4'-Tetrahydroxystilbene-2-*O-*β*-D*-glucoyranoside | Cis-2,3,5,4'-tetrahydroxystilbene-2-*O-*β-*D*-glucoyranoside | 2,3,5,4'-Tetrahydroxystilbene-2-*O*-(2”-*O*-feruloyl)-β*-D*-glucopyranoside | Polydatin | Resveratrol |
| --- | --- | --- | --- | --- | --- |
| S1 | 4089.14 | 122.54 | 35.84 | 13.61 | 92.39 |
| S2 | 4427.64 | 191.72 | 24.48 | 11.58 | 55.48 |
| S3 | 4966.56 | 145.09 | 34.47 | 8.98 | 99.60 |
| S4 | 6475.38 | 456.87 | 29.83 | 7.87 | 114.72 |
| S5 | 4497.28 | 293.69 | 30.90 | 15.74 | 108.53 |
| S6 | 29646.80 | 647.16 | 135.20 | 74.65 | 383.89 |
| S7 | 43266.12 | 600.96 | 150.12 | 68.71 | 466.10 |
| S8 | 36056.03 | 584.78 | 146.60 | 105.60 | 527.46 |
| S9 | 40075.91 | 538.55 | 174.21 | 54.43 | 417.00 |
| S10 | 42729.42 | 721.99 | 183.16 | 70.89 | 444.82 |
| S11 | 20678.86 | 193.46 | 216.89 | 50.88 | 630.68 |
| S12 | 18722.21 | 423.37 | 108.13 | 25.62 | 350.75 |
| S13 | 20567.85 | 264.56 | 128.28 | 19.50 | 356.65 |
| S14 | 20957.55 | 550.57 | 103.96 | 29.10 | 198.84 |
| S15 | 22871.48 | 581.96 | 120.58 | 27.21 | 195.86 |
| S16 | 20827.63 | 684.81 | 116.29 | 33.44 | 405.27 |
| S17 | 21241.62 | 334.06 | 103.39 | 29.87 | 243.11 |
| S18 | 22479.65 | 317.84 | 116.66 | 29.60 | 437.05 |
| S19 | 18360.68 | 338.39 | 121.12 | 26.27 | 278.11 |
| S20 | 30016.06 | 426.58 | 169.75 | 43.25 | 510.97 |
| S21 | 29003.12 | 313.25 | 153.19 | 27.61 | 481.96 |
| S22 | 23069.86 | 159.56 | 119.74 | 46.59 | 424.90 |
| S23 | 21732.40 | 207.46 | 108.89 | 50.58 | 317.31 |
| S24 | 20418.87 | 196.77 | 109.75 | 31.53 | 519.29 |
| S25 | 20072.53 | 303.27 | 105.22 | 38.07 | 355.27 |
| S26 | 20271.10 | 223.00 | 104.08 | 34.33 | 340.99 |
| S27 | 38384.30 | 593.80 | 178.34 | 175.02 | 225.64 |
| S28 | 28380.07 | 396.29 | 63.16 | 52.67 | 104.61 |
| S29 | 26566.11 | 759.52 | 62.88 | 53.92 | 45.61 |
| S30 | 25049.19 | 758.87 | 64.51 | 56.67 | 56.43 |
| S31 | 38041.12 | 521.28 | 180.82 | 116.72 | 142.98 |
| S32 | 36812.31 | 741.96 | 221.59 | 107.17 | 121.91 |
| S33 | 49058.84 | 693.91 | 231.54 | 125.65 | 97.57 |
| S34 | 43540.65 | 482.84 | 179.55 | 132.86 | 132.08 |
| S35 | 47611.23 | 510.55 | 194.82 | 199.49 | 112.36 |
| S36 | 41133.85 | 578.05 | 263.90 | 120.86 | 129.46 |
| S37 | 45409.51 | 687.31 | 252.42 | 103.34 | 136.71 |
| S38 | 33243.29 | 796.58 | 240.94 | 85.81 | 143.95 |
| S39 | 54994.38 | 672.16 | 233.19 | 96.74 | 163.12 |
| S40 | 57243.12 | 608.67 | 227.49 | 873.26 | 477.28 |
| S41 | 47076.85 | 607.65 | 266.57 | 96.16 | 108.97 |
| S42 | 45445.15 | 622.87 | 179.79 | 70.31 | 64.51 |
| S43 | 41092.01 | 819.49 | 260.43 | 98.06 | 90.45 |
| S44 | 41306.75 | 711.48 | 321.29 | 98.03 | 85.40 |
| S45 | 32851.20 | 822.41 | 304.06 | 89.08 | 79.55 |
| S46 | 43309.30 | 663.30 | 305.99 | 100.32 | 99.80 |
| S47 | 39157.94 | 680.70 | 286.01 | 103.47 | 101.83 |
| S48 | 43830.71 | 714.76 | 255.07 | 81.39 | 89.72 |
| S49 | 43796.65 | 884.72 | 351.33 | 100.60 | 128.38 |
| S50 | 41938.58 | 933.50 | 324.77 | 111.05 | 108.53 |
| S51 | 34633.14 | 104.99 | 121.62 | 180.72 | 371.07 |
| S52 | 21504.84 | 250.35 | 71.35 | 70.87 | 398.97 |
| S53 | 20607.12 | 197.58 | 101.55 | 55.22 | 491.16 |
| S54 | 21741.71 | 348.46 | 77.91 | 94.32 | 722.86 |
| S55 | 30972.98 | 110.94 | 86.05 | 168.09 | 746.76 |
| S56 | 18172.97 | 227.11 | 57.69 | 63.38 | 538.80 |
| S57 | 15377.66 | 397.21 | 68.03 | 137.38 | 799.14 |
| S58 | 25782.81 | 225.41 | 72.45 | 82.85 | 589.17 |
| S59 | 23288.17 | 281.45 | 64.58 | 65.61 | 609.60 |
| S60 | 18194.71 | 256.38 | 63.92 | 76.03 | 611.84 |
| S61 | 28460.14 | 165.05 | 109.19 | 69.92 | 606.79 |

**Table S5.** Concentrations of flavonoids and phenols in PM (µg/g).

| Sample | Catechin | Epicatechin | Hyperoside | Rutin | Gallic acid | *p*-Hydroxy-benzoic acid | *p*-Hydroxyben-zaldehyde | *p*-Coumaric acid | Torachrysone 8-*O*-glucoside |
| --- | --- | --- | --- | --- | --- | --- | --- | --- | --- |
| S1 | 2060.02 | 124.52 | 0.54 | 1.02 | 41.64 | 1.38 | 4.76 | 1.73 | 11.97 |
| S2 | 2105.29 | 188.46 | 0.97 | 1.72 | 162.61 | 1.45 | 4.38 | 2.22 | 2.46 |
| S3 | 1820.93 | 121.78 | 0.75 | 1.80 | 41.94 | 1.25 | 4.30 | 2.15 | 7.80 |
| S4 | 2015.95 | 153.74 | 1.24 | 1.70 | 77.34 | 1.27 | 4.56 | 2.12 | 8.70 |
| S5 | 1949.08 | 168.82 | 1.51 | 1.98 | 60.18 | 1.48 | 3.81 | 2.56 | 8.97 |
| S6 | 596.31 | 75.65 | 0.06 | 0.26 | 73.45 | 3.67 | 18.96 | 2.17 | 318.72 |
| S7 | 531.63 | 88.41 | 0.06 | 0.37 | 69.58 | 4.52 | 21.46 | 2.14 | 259.41 |
| S8 | 730.53 | 90.12 | 0.09 | 0.83 | 56.18 | 4.64 | 21.29 | 3.09 | 298.95 |
| S9 | 675.28 | 101.19 | 0.18 | 0.54 | 63.62 | 6.33 | 20.33 | 2.72 | 294.00 |
| S10 | 670.03 | 89.60 | 0.11 | 0.66 | 57.81 | 6.43 | 20.96 | 2.52 | 321.71 |
| S11 | 1184.68 | 53.90 | 0.00 | 0.14 | 136.48 | 1.47 | 7.39 | 3.30 | 44.27 |
| S12 | 423.26 | 178.27 | 0.82 | 0.20 | 83.26 | 4.79 | 21.24 | 6.01 | 98.91 |
| S13 | 456.63 | 197.27 | 1.09 | 0.20 | 93.89 | 5.65 | 19.57 | 4.97 | 141.36 |
| S14 | 733.71 | 300.04 | 0.35 | 0.18 | 82.69 | 2.15 | 13.61 | 3.55 | 200.34 |
| S15 | 785.41 | 281.28 | 0.51 | 0.16 | 74.62 | 2.60 | 13.40 | 4.02 | 183.90 |
| S16 | 844.75 | 264.26 | 0.65 | 0.19 | 77.65 | 3.31 | 15.03 | 3.99 | 185.94 |
| S17 | 838.90 | 241.08 | 0.27 | 0.13 | 67.23 | 1.90 | 12.68 | 3.47 | 174.52 |
| S18 | 708.47 | 269.43 | 0.76 | 0.14 | 80.88 | 3.03 | 13.04 | 4.05 | 170.23 |
| S19 | 906.00 | 283.01 | 0.51 | 0.15 | 77.63 | 2.76 | 12.67 | 3.74 | 168.05 |
| S20 | 254.96 | 157.99 | 0.84 | 0.25 | 198.38 | 8.88 | 38.01 | 5.04 | 59.34 |
| S21 | 206.90 | 161.82 | 0.88 | 0.20 | 287.60 | 4.85 | 20.37 | 4.50 | 48.56 |
| S22 | 1669.17 | 79.53 | 0.00 | 0.17 | 149.39 | 1.65 | 13.77 | 3.86 | 123.94 |
| S23 | 1419.68 | 77.72 | 0.00 | 0.16 | 127.01 | 1.60 | 13.01 | 2.70 | 151.75 |
| S24 | 1458.34 | 68.34 | 0.00 | 0.13 | 123.77 | 1.81 | 11.92 | 2.57 | 90.39 |
| S25 | 1343.84 | 67.41 | 0.00 | 0.13 | 115.00 | 1.60 | 11.02 | 2.73 | 116.63 |
| S26 | 1375.68 | 64.52 | 0.00 | 0.15 | 123.03 | 2.11 | 13.52 | 2.62 | 89.72 |
| S27 | 1398.32 | 169.31 | 0.00 | 0.78 | 126.96 | 2.52 | 18.41 | 1.73 | 164.89 |
| S28 | 814.30 | 38.71 | 0.00 | 0.00 | 44.29 | 1.83 | 4.29 | 0.47 | 161.48 |
| S29 | 818.23 | 44.90 | 0.00 | 0.00 | 56.46 | 1.60 | 6.50 | 0.42 | 158.70 |
| S30 | 758.37 | 45.23 | 0.00 | 0.00 | 61.47 | 1.76 | 7.07 | 0.80 | 179.54 |
| S31 | 1388.53 | 234.65 | 0.11 | 0.27 | 28.96 | 1.42 | 7.58 | 0.00 | 284.73 |
| S32 | 1724.05 | 262.25 | 0.07 | 0.27 | 44.97 | 1.29 | 8.70 | 0.00 | 273.83 |
| S33 | 2068.20 | 266.35 | 0.06 | 0.23 | 33.18 | 1.29 | 8.32 | 0.00 | 355.32 |
| S34 | 1662.37 | 315.54 | 0.20 | 0.46 | 32.96 | 1.61 | 8.54 | 0.00 | 338.06 |
| S35 | 1891.86 | 367.04 | 0.12 | 0.37 | 24.69 | 1.36 | 8.62 | 0.00 | 317.49 |
| S36 | 2088.95 | 244.87 | 0.08 | 0.27 | 42.62 | 1.31 | 7.54 | 0.00 | 279.52 |
| S37 | 1962.72 | 248.84 | 0.13 | 0.13 | 46.90 | 1.06 | 6.47 | 0.00 | 266.06 |
| S38 | 1836.48 | 252.80 | 0.19 | 0.26 | 51.18 | 1.95 | 8.82 | 0.00 | 252.60 |
| S39 | 2044.92 | 234.01 | 0.06 | 0.31 | 43.78 | 1.79 | 9.36 | 0.00 | 335.03 |
| S40 | 2126.87 | 261.25 | 0.15 | 0.39 | 33.73 | 1.40 | 8.03 | 0.00 | 467.32 |
| S41 | 2304.12 | 321.93 | 0.09 | 0.37 | 28.92 | 1.28 | 7.20 | 0.00 | 325.47 |
| S42 | 1771.01 | 370.43 | 0.10 | 0.41 | 25.90 | 1.37 | 5.97 | 0.00 | 347.77 |
| S43 | 2264.86 | 274.24 | 0.08 | 0.33 | 34.16 | 1.30 | 6.28 | 0.00 | 278.87 |
| S44 | 2144.35 | 302.58 | 0.09 | 0.41 | 35.22 | 1.16 | 6.69 | 0.00 | 247.34 |
| S45 | 2245.34 | 271.82 | 0.07 | 0.26 | 28.86 | 1.26 | 6.20 | 0.00 | 243.71 |
| S46 | 2528.54 | 288.30 | 0.06 | 0.35 | 32.26 | 1.14 | 5.91 | 0.00 | 265.60 |
| S47 | 1807.72 | 311.94 | 0.08 | 0.35 | 24.05 | 1.29 | 5.89 | 0.00 | 304.74 |
| S48 | 2260.56 | 332.17 | 0.10 | 0.32 | 23.07 | 1.54 | 6.67 | 0.00 | 305.23 |
| S49 | 2200.54 | 241.22 | 0.09 | 0.35 | 32.50 | 1.33 | 7.34 | 0.00 | 347.92 |
| S50 | 1803.67 | 259.87 | 0.07 | 0.37 | 25.12 | 1.30 | 6.66 | 0.00 | 296.01 |
| S51 | 115.90 | 97.91 | 0.14 | 1.28 | 190.59 | 6.40 | 30.08 | 3.88 | 138.15 |
| S52 | 473.43 | 189.45 | 0.09 | 0.49 | 106.19 | 2.60 | 12.99 | 4.07 | 206.62 |
| S53 | 419.74 | 118.39 | 0.13 | 0.50 | 148.39 | 6.16 | 13.50 | 6.27 | 172.38 |
| S54 | 277.23 | 208.07 | 0.15 | 0.48 | 190.07 | 6.45 | 15.14 | 5.84 | 134.61 |
| S55 | 96.43 | 87.73 | 0.13 | 0.97 | 189.54 | 6.01 | 22.78 | 2.68 | 131.07 |
| S56 | 445.48 | 112.56 | 0.13 | 0.57 | 110.78 | 3.58 | 17.95 | 5.14 | 177.08 |
| S57 | 270.95 | 100.14 | 0.09 | 0.24 | 150.16 | 4.25 | 11.42 | 3.13 | 76.34 |
| S58 | 477.51 | 143.59 | 0.15 | 0.53 | 88.82 | 4.74 | 18.09 | 5.15 | 194.27 |
| S59 | 447.11 | 155.07 | 0.16 | 0.58 | 97.84 | 4.66 | 17.76 | 4.97 | 164.67 |
| S60 | 583.36 | 135.92 | 0.12 | 0.31 | 93.33 | 4.47 | 16.16 | 6.96 | 95.47 |
| S61 | 677.97 | 77.30 | 0.19 | 0.99 | 85.72 | 3.65 | 30.87 | 5.38 | 301.27 |

**Table S6.** Cell viability (%) and the half inhibitory concentration (IC_50_) of PM.

| Sample No. | Concentration (μg/mL) | SD (%) | Cell Viability (%) | IC_50_ ± SD |  |
| --- | --- | --- | --- | --- | --- |
| S1 | | 1 | 3.4186 | 105.6084 | 537 ± 22 |
|  |  | 10 | 4.7914 | 102.2875 |  |
|  |  | 100 | 2.7440 | 95.3132 |  |
|  |  | 1000 | 2.4919 | 24.5872 |  |
| S3 | | 1 | 3.4310 | 102.6146 | 540± 24 |
|  |  | 10 | 2.5646 | 100.2654 |  |
|  |  | 100 | 1.3651 | 97.7930 |  |
|  |  | 1000 | 5.5919 | 22.2762 |  |
| S6 | 1 | 4.6832 | 108.7949 | 419 ± 32 |  |
|  | 10 | 4.5879 | 99.8044 |  |  |
|  | 100 | 4.3358 | 89.8774 |  |  |
|  | 1000 | 2.3403 | 24.8150 |  |  |
| S8 | 1 | 5.3198 | 110.8358 | 405 ± 30 |  |
|  | 10 | 2.2921 | 99.1104 |  |  |
|  | 100 | 5.9588 | 89.2988 |  |  |
|  | 1000 | 1.1603 | 22.4116 |  |  |
| S9 | 1 | 3.2649 | 103.3425 | 416 ± 20 |  |
|  | 10 | 5.2167 | 96.4429 |  |  |
|  | 100 | 1.6227 | 85.1422 |  |  |
|  | 1000 | 2.2774 | 24.1471 |  |  |
| S11 | 1 | 3.5321 | 105.9322 | 502 ± 39 |  |
|  | 10 | 4.3197 | 100.1481 |  |  |
|  | 100 | 1.7120 | 95.6181 |  |  |
|  | 1000 | 3.3532 | 19.8532 |  |  |
| S12 | 1 | 8.0168 | 105.4339 | 494 ± 14 |  |
|  | 10 | 6.3045 | 102.7533 |  |  |
|  | 100 | 4.8972 | 96.4247 |  |  |
|  | 1000 | 2.3995 | 23.8525 |  |  |
| S14 | 1 | 2.2977 | 106.1613 | 497 ± 29 |  |
|  | 10 | 6.5786 | 101.1384 |  |  |
|  | 100 | 3.8602 | 97.2976 |  |  |
|  | 1000 | 1.8675 | 21.2538 |  |  |
| S18 | 1 | 4.7539 | 103.6075 | 499 ± 36 |  |
|  | 10 | 6.0241 | 98.2310 |  |  |
|  | 100 | 4.3062 | 95.1493 |  |  |
|  | 1000 | 1.2382 | 20.4771 |  |  |
| S19 | 1 | 6.2026 | 105.5524 | 496 ± 28 |  |
|  | 10 | 2.3935 | 100.4509 |  |  |
|  | 100 | 4.3957 | 96.5865 |  |  |
|  | 1000 | 1.8663 | 21.2399 |  |  |
| S21 | 1 | 6.4238 | 102.0786 | 398 ± 11 |  |
|  | 10 | 5.5000 | 100.3934 |  |  |
|  | 100 | 4.9130 | 81.3804 |  |  |
|  | 1000 | 0.5814 | 20.0775 |  |  |
| S22 | 1 | 5.1909 | 103.6645 | 505 ± 24 |  |
|  | 10 | 4.1826 | 101.9425 |  |  |
|  | 100 | 4.3873 | 96.2231 |  |  |
|  | 1000 | 2.8169 | 21.6126 |  |  |
| S25 | 1 | 3.8479 | 100.5296 | 505 ± 33 |  |
|  | 10 | 4.0771 | 97.4224 |  |  |
|  | 100 | 5.0763 | 92.9637 |  |  |
|  | 1000 | 4.3573 | 17.9456 |  |  |
| S27 | 1 | 3.1900 | 95.6737 | 383 ± 43 |  |
|  | 10 | 6.4344 | 89.9415 |  |  |
|  | 100 | 5.3099 | 75.0476 |  |  |
|  | 1000 | 1.1674 | 19.3910 |  |  |
| S28 | 1 | 2.9432 | 100.1631 | 485 ± 20 |  |
|  | 10 | 2.0067 | 97.7916 |  |  |
|  | 100 | 5.0314 | 89.9311 |  |  |
|  | 1000 | 2.4684 | 19.5775 |  |  |
| S30 | 1 | 4.3219 | 101.1046 | 492 ± 31 |  |
|  | 10 | 5.5094 | 99.0503 |  |  |
|  | 100 | 4.4950 | 92.2682 |  |  |
|  | 1000 | 3.6283 | 23.3755 |  |  |
| S31 | 1 | 8.2669 | 104.8844 | 357 ± 29 |  |
|  | 10 | 3.4274 | 96.8788 |  |  |
|  | 100 | 6.1892 | 79.7546 |  |  |
|  | 1000 | 1.3413 | 21.3006 |  |  |
| S33 | 1 | 6.6435 | 106.5248 | 346 ± 11 |  |
|  | 10 | 3.7677 | 96.7634 |  |  |
|  | 100 | 4.4808 | 79.4544 |  |  |
|  | 1000 | 1.4663 | 19.7333 |  |  |
| S34 | 1 | 3.3639 | 101.2507 | 352 ± 19 |  |
|  | 10 | 4.5206 | 88.8236 |  |  |
|  | 100 | 3.8060 | 76.7782 |  |  |
|  | 1000 | 1.8273 | 21.2315 |  |  |
| S38 | 1 | 4.0634 | 99.8065 | 348 ± 21 |  |
|  | 10 | 6.2290 | 89.1872 |  |  |
|  | 100 | 3.9539 | 74.9543 |  |  |
|  | 1000 | 2.2508 | 19.6590 |  |  |
| S39 | 1 | 3.6511 | 100.6726 | 340 ± 36 |  |
|  | 10 | 5.4630 | 88.1585 |  |  |
|  | 100 | 5.7965 | 74.8957 |  |  |
|  | 1000 | 2.9262 | 19.5717 |  |  |
| S40 | 1 | 2.0882 | 98.4191 | 353 ± 24 |  |
|  | 10 | 3.9067 | 90.0574 |  |  |
|  | 100 | 5.0654 | 74.3985 |  |  |
|  | 1000 | 2.9420 | 19.6643 |  |  |
| S41 | 1 | 5.1806 | 100.2362 | 346 ± 19 |  |
|  | 10 | 4.9814 | 92.3537 |  |  |
|  | 100 | 2.9265 | 75.2959 |  |  |
|  | 1000 | 3.5087 | 20.2640 |  |  |
| S45 | 1 | 3.9995 | 102.7125 | 342 ± 33 |  |
|  | 10 | 7.2919 | 92.5866 |  |  |
|  | 100 | 3.1350 | 76.8072 |  |  |
|  | 1000 | 4.3520 | 20.6978 |  |  |
| S46 | 1 | 5.0294 | 101.0673 | 350 ± 32 |  |
|  | 10 | 5.2164 | 88.3086 |  |  |
|  | 100 | 4.2497 | 76.0154 |  |  |
|  | 1000 | 3.7997 | 19.6190 |  |  |
| S48 | 1 | 3.5451 | 99.6707 | 351 ± 27 |  |
|  | 10 | 5.2820 | 91.4305 |  |  |
|  | 100 | 4.8586 | 75.6031 |  |  |
|  | 1000 | 4.0030 | 21.1043 |  |  |
| S52 | 1 | 6.9961 | 103.9674 | 395 ± 35 |  |
|  | 10 | 3.8607 | 99.5342 |  |  |
|  | 100 | 5.8816 | 81.2036 |  |  |
|  | 1000 | 3.3378 | 20.9276 |  |  |
| S54 | 1 | 3.0182 | 101.7704 | 412 ± 9 |  |
|  | 10 | 1.7014 | 99.9456 |  |  |
|  | 100 | 4.5532 | 82.8859 |  |  |
|  | 1000 | 2.2227 | 21.2206 |  |  |
| S58 | 1 | 2.2385 | 100.5709 | 418 ± 17 |  |
|  | 10 | 3.7212 | 97.6095 |  |  |
|  | 100 | 5.8645 | 82.6085 |  |  |
|  | 1000 | 3.7094 | 21.4384 |  |  |
| S60 | 1 | 4.8601 | 101.5783 | 420 ± 24 |  |
|  | 10 | 5.7048 | 96.5934 |  |  |
|  | 100 | 5.4950 | 84.2036 |  |  |
|  | 1000 | 1.0546 | 24.2815 |  |  |
| S61 | 1 | 2.7266 | 105.4538 | 409 ± 31 |  |
|  | 10 | 4.9169 | 99.9226 |  |  |
|  | 100 | 4.9179 | 85.3870 |  |  |
|  | 1000 | 2.9062 | 21.5302 |  |  |

**Table S7.** The chromaticity values of the four parts of PM decoction pieces: (1) The cork site. (2) The front and back of the phloem par. (3) The front and back of the central xylem. (4) The front and back of the heteromorphic vascular bundles.

| Sample No. | S11 | S14 | S18 | S22 | S28 | S27 | S31 | S38 | S41 | S46 | S8 | S9 | S52 | S58 | S60 |
| --- | --- | --- | --- | --- | --- | --- | --- | --- | --- | --- | --- | --- | --- | --- | --- |
| R (1) | 109.6 | 107.8 | 100.2 | 100.2 | 108.0 | 129.2 | 129.6 | 119.0 | 109.8 | 99.6 | 70.6 | 83.4 | 91.4 | 102.0 | 94.2 |
| G (1) | 112.8 | 104.6 | 93.4 | 95.2 | 103.8 | 107.0 | 119.0 | 108.4 | 105.0 | 96.4 | 72.4 | 84.6 | 93.8 | 95.4 | 96.6 |
| B (1) | 120.6 | 109.4 | 89.6 | 94.0 | 106.4 | 99.0 | 114.4 | 107.0 | 86.2 | 100.0 | 73.4 | 84.4 | 100.8 | 92.6 | 98.0 |
| L*(1) | 47.4 | 44.8 | 40.0 | 42.0 | 44.4 | 45.2 | 50.8 | 46.6 | 44.8 | 41.0 | 30.2 | 36.0 | 39.8 | 39.6 | 40.6 |
| a*(1) | 0.2 | 2.4 | 2.2 | 1.0 | 1.8 | 2.8 | 3.4 | 4.2 | 2.2 | 1.8 | -0.6 | -0.2 | 0.2 | 3.4 | -0.6 |
| b*(1) | -4.4 | -2.0 | 3.2 | 1.2 | -1.8 | 2.2 | 4.0 | 2.4 | 0.0 | 0.2 | -1.0 | 0.0 | -4.4 | 3.4 | -1.4 |
| E*ab (1) | 47.8 | 45.2 | 40.4 | 42.6 | 44.7 | 45.5 | 51.3 | 47.1 | 45.2 | 41.3 | 30.5 | 36.4 | 40.3 | 40.2 | 40.8 |
| R(2) | 201.8 | 213.1 | 196.4 | 194.7 | 213.3 | 204.0 | 209.6 | 206.8 | 202.2 | 212.3 | 174.7 | 155.4 | 166.7 | 173.6 | 175.2 |
| G(2) | 204.3 | 223.4 | 207.4 | 204.9 | 220.1 | 204.1 | 202.6 | 198.7 | 204.5 | 202.3 | 175.1 | 152.3 | 160.1 | 175.4 | 167.9 |
| B(2) | 203.3 | 208.7 | 197.5 | 193.9 | 204.9 | 170.6 | 199.0 | 159.7 | 173.6 | 175.3 | 159.4 | 136.8 | 137.1 | 173.4 | 150.0 |
| L*(2) | 84.3 | 87.9 | 82.3 | 81.5 | 89.0 | 74.0 | 82.0 | 80.1 | 69.7 | 81.8 | 71.2 | 62.7 | 65.9 | 71.5 | 69.0 |
| a*(2) | -5.3 | -5.5 | -5.3 | -5.1 | -5.5 | -0.4 | 2.2 | -1.6 | -0.2 | 0.2 | -2.2 | -0.9 | -0.5 | -0.9 | 0.5 |
| b*(2) | 3.7 | 6.0 | 3.0 | 3.7 | 5.4 | 6.2 | 2.8 | 20.7 | 3.7 | 15.0 | 8.2 | 8.5 | 13.0 | 0.9 | 10.3 |
| E*ab(2) | 84.7 | 88.6 | 82.7 | 81.9 | 89.3 | 74.5 | 82.3 | 82.8 | 69.9 | 83.2 | 71.8 | 63.6 | 67.7 | 71.5 | 69.8 |
| R(3) | 190.7 | 206.0 | 209.5 | 210.8 | 204.8 | 205.5 | 213.7 | 214.4 | 199.0 | 210.5 | 159.4 | 184.1 | 184.1 | 186.0 | 185.3 |
| G(3) | 211.3 | 217.3 | 217.9 | 219.2 | 217.7 | 190.2 | 202.9 | 210.9 | 178.7 | 209.6 | 156.2 | 181.7 | 182.1 | 188.8 | 176.2 |
| B(3) | 215.7 | 218.7 | 210.6 | 214.3 | 218.9 | 191.8 | 195.8 | 189.4 | 193.0 | 193.5 | 135.9 | 182.0 | 160.7 | 196.4 | 158.0 |
| L*(3) | 83.1 | 86.0 | 85.8 | 86.9 | 86.0 | 77.2 | 82.4 | 84.2 | 72.8 | 83.9 | 64.3 | 74.0 | 73.8 | 76.6 | 72.4 |
| a*(3) | -6.6 | -3.7 | -4.1 | -3.6 | -4.3 | 0.5 | 3.1 | -1.3 | -0.5 | -1.5 | -1.5 | 0.9 | -1.9 | 0.2 | 1.0 |
| b*(3) | -3.6 | -2.2 | 1.9 | 2.8 | -2.2 | 2.9 | 5.0 | 11.1 | -1.1 | 7.8 | 11.1 | 0.2 | 11.2 | -4.3 | 10.6 |
| E*ab(3) | 83.4 | 86.1 | 85.9 | 87.0 | 86.1 | 77.3 | 82.6 | 85.0 | 72.8 | 84.3 | 65.4 | 74.0 | 74.7 | 76.7 | 73.2 |
| R(4) | 189.9 | 204.1 | 203.7 | 205.9 | 201.7 | 210.3 | 218.2 | 216.9 | 201.8 | 220.1 | 193.6 | 178.5 | 192.1 | 175.7 | 182.3 |
| G(4) | 212.9 | 219.7 | 218.9 | 217.6 | 218.7 | 208.0 | 213.3 | 212.3 | 195.2 | 213.1 | 192.5 | 179.4 | 195.4 | 173.0 | 175.3 |
| B(4) | 215.5 | 221.9 | 215.1 | 212.5 | 221.5 | 203.8 | 207.5 | 184.7 | 198.9 | 198.0 | 177.4 | 179.4 | 178.0 | 167.7 | 157.1 |
| L*(4) | 83.4 | 86.6 | 85.5 | 85.9 | 86.1 | 83.3 | 85.1 | 84.9 | 78.4 | 84.8 | 77.6 | 72.9 | 78.1 | 70.9 | 71.7 |
| a*(4) | -7.5 | -5.3 | -5.0 | -4.8 | -5.6 | -1.0 | 1.1 | -1.6 | -2.1 | -0.6 | -1.5 | -0.2 | -3.3 | 0.2 | 0.2 |
| b*(4) | -4.0 | -2.9 | 0.9 | 1.2 | -3.4 | 4.4 | 4.0 | 14.4 | -0.4 | 8.1 | 7.7 | 0.0 | 8.5 | 3.1 | 10.4 |
| E*ab(4) | 83.8 | 86.8 | 85.7 | 86.0 | 86.3 | 83.4 | 85.2 | 86.1 | 78.4 | 85.2 | 78.0 | 72.9 | 78.6 | 71.0 | 72.5 |
